# Reduced dengue incidence following city-wide *w*Mel *Wolbachia* mosquito releases throughout three Colombian cities: Interrupted time series analysis and a prospective case-control study

**DOI:** 10.1371/journal.pntd.0011713

**Published:** 2023-11-30

**Authors:** Ivan Dario Velez, Stephanie K. Tanamas, Maria Patricia Arbelaez, Simon C. Kutcher, Sandra L. Duque, Alexander Uribe, Lina Zuluaga, Luis Martínez, Ana Cristina Patiño, Jovany Barajas, Estefanía Muñoz, Maria Camila Mejia Torres, Sandra Uribe, Sandra Porras, Rita Almanza, Henry Pulido, Scott L. O’Neill, Eduardo Santacruz-Sanmartin, Sandra Gonzalez, Peter A. Ryan, Jai A. Denton, Nicholas P. Jewell, Suzanne M. Dufault, Cameron P. Simmons, Katherine L. Anders

**Affiliations:** 1 World Mosquito Program, Universidad de Antioquia, Medellín, Colombia; 2 World Mosquito Program, Monash University, Melbourne, Australia; 3 Secretariat of Health, Medellín, Colombia; 4 Secretariat of Health, Bello, Colombia; 5 Department of Medical Statistics, London School of Hygiene and Tropical Medicine, London, United Kingdom; 6 Division of Biostatistics, Department of Epidemiology and Biostatistics, University of California, San Francisco, California, United States of America; Australian Red Cross Lifelood, AUSTRALIA

## Abstract

**Background:**

The introduction of *Wolbachia* (*w*Mel strain) into *Aedes aegypti* mosquitoes reduces their capacity to transmit dengue and other arboviruses. Randomised and non-randomised studies in multiple countries have shown significant reductions in dengue incidence following field releases of *w*Mel-infected *Ae*. *aegypti*. We report the public health outcomes from phased, large-scale releases of *w*Mel-*Ae*. *aegypti* mosquitoes throughout three contiguous cities in the Aburrá Valley, Colombia.

**Methodology/Principal findings:**

Following pilot releases in 2015–2016, staged city-wide *w*Mel-*Ae*. *aegypti* deployments were undertaken in the cities of Bello, Medellín and Itagüí (3.3 million people) between October 2016 and April 2022. The impact of the *Wolbachia* intervention on dengue incidence was evaluated in two parallel studies. A quasi-experimental study using interrupted time series analysis showed notified dengue case incidence was reduced by 95% in Bello and Medellín and 97% in Itagüí, following establishment of *w*Mel at ≥60% prevalence, compared to the pre-intervention period and after adjusting for seasonal trends. A concurrent clinic-based case-control study with a test-negative design was unable to attain the target sample size of 63 enrolled virologically-confirmed dengue (VCD) cases between May 2019 and December 2021, consistent with low dengue incidence throughout the Aburrá Valley following *w*Mel deployments. Nevertheless, VCD incidence was 45% lower (OR 0.55 [95% CI 0.25, 1.17]) and combined VCD/presumptive dengue incidence was 47% lower (OR 0.53 [95% CI 0.30, 0.93]) among participants resident in *w*Mel-treated versus untreated neighbourhoods.

**Conclusions/Significance:**

Stable introduction of *w*Mel into local *Ae*. *aegypti* populations was associated with a significant and sustained reduction in dengue incidence across three Colombian cities. These results from the largest contiguous *Wolbachia* releases to-date demonstrate the real-world effectiveness of the method across large urban populations and, alongside previously published results, support the reproducibility of this effectiveness across different ecological settings.

**Trial registration:**

NCT03631719.

## Author summary

Dengue fever is a viral disease transmitted by *Aedes aegypti* mosquitoes and is an increasing public health concern globally. A novel evidence-based tool for dengue control involves the release of *Ae*. *aegypti* mosquitoes infected with a naturally-occurring insect bacteria called *Wolbachia* (*w*Mel strain). *w*Mel significantly reduces the mosquito’s ability to transmit dengue and other viruses and can establish long-term in local *Ae*. *aegypti* populations following short-term releases. Previous studies have shown that establishment of *w*Mel in local mosquito populations significantly reduces local dengue incidence. In Colombia, *w*Mel-infected *Ae*. *aegypti* were released throughout the cities of Bello, Medellín, and Itagüí in the Aburrá Valley between 2015 and 2022, covering a population of 3.3 million people. The rate of dengue case notifications in the three cities declined by 95–97% after *Wolbachia* releases, compared to the prior decade, and dengue case numbers since 2020 have been the lowest in twenty years. A case-control study in Medellin in 2019–2021 showed that laboratory-confirmed dengue incidence was significantly lower in neighbourhoods with *Wolbachia* releases compared to untreated neighbourhoods. These results confirm the real-world effectiveness of the *Wolbachia* method, and show that it can be implemented at a city-wide scale to protect communities against dengue.

## Introduction

Dengue is a growing global health challenge, with climate change and urbanisation driving an increase in the population vulnerable to dengue epidemics [1,2]. Latin America has seen the greatest relative increase in dengue disease burden over the past two decades [3]. The primary vector for dengue, the *Aedes aegypti* mosquito, also transmits the chikungunya and Zika viruses, both of which have been circulating in Colombia since their first detection in 2014 and 2015 respectively [4,5].

The introduction of the insect bacterium *Wolbachia* (*w*Mel strain) into *Ae*. *aegypti* mosquitoes reduces their ability to transmit human pathogens including dengue, Zika, chikungunya, and yellow fever [6–8]. *Wolbachia* is maternally inherited through successive generations and manipulates insect reproduction to favour its own population dissemination through a process of cytoplasmic incompatibility [8,9]. These characteristics facilitate its application as a public health tool, delivered as short-term releases of *w*Mel-infected *Ae*. *aegypti* into residential areas which drives introgression of *w*Mel into local *Ae*. *aegypti* populations [10], resulting in a mosquito population that is refractory to local dengue virus transmission. The feasibility, acceptability, effectiveness and durability of the *Wolbachia* method has been demonstrated in numerous field trials in Asia-Pacific and Latin American countries [11–19]. A cluster randomised efficacy trial in Yogyakarta, Indonesia, demonstrated a 77% reduction in the incidence of virologically-confirmed dengue and an 86% reduction in dengue hospitalisations in *Wolbachia*-treated versus untreated areas [14]. These results, together with consistent findings from non-randomised *w*Mel deployments in multiple countries [11–13,15–17], led the Vector Control Advisory Group of the World Health Organisation to endorse the evidence for *Wolbachia* as an effective method of dengue control [20]. To date, *w*Mel-infected *Ae*. *aegypti* have been deployed in communities in 11 countries, reaching an estimated 11 million people.

In Colombia, pilot *Wolbachia* deployments were undertaken in several neighbourhoods in the municipality of Bello in 2015–2016. The declaration of Zika as a public health emergency by the WHO in early 2016 [21] accelerated the planned expansion of pilot releases to city-scale, with the aim of optimising methods for scaled deployment under operational conditions while also evaluating the epidemiological effectiveness against *Aedes-*borne viruses [22]. We report here the public health outcomes of city-wide deployments of *w*Mel-infected *Ae*. *aegypti* mosquitoes throughout the adjacent municipalities of Bello, Medellín and Itagüí in the Aburrá Valley in Colombia, which represent the largest contiguous implementation of the *Wolbachia* method to date. The entomological outcomes of the deployments are reported in a concurrent publication [23]. The impact of the *Wolbachia* intervention on dengue incidence was evaluated in two parallel studies. A quasi-experimental study in all three municipalities used interrupted time series analysis to quantify the reduction in the incidence of dengue cases notified to the routine disease surveillance system. In parallel, a prospective clinic-based case-control study was conducted in one quadrant of Medellín to evaluate the impact of the *w*Mel deployment on the incidence of virologically-confirmed dengue.

## Methods

### Ethics statement

The protocol for the case-control study was approved by the human research ethics committees of Universidad de Antioquia and Monash University, and has been published previously [22]; ClinicalTrials.gov identifier NCT03631719]. Written informed consent was obtained from all participants, or from their guardian where a participant was under 18 years of age.

### Study setting

**Fig 1:**
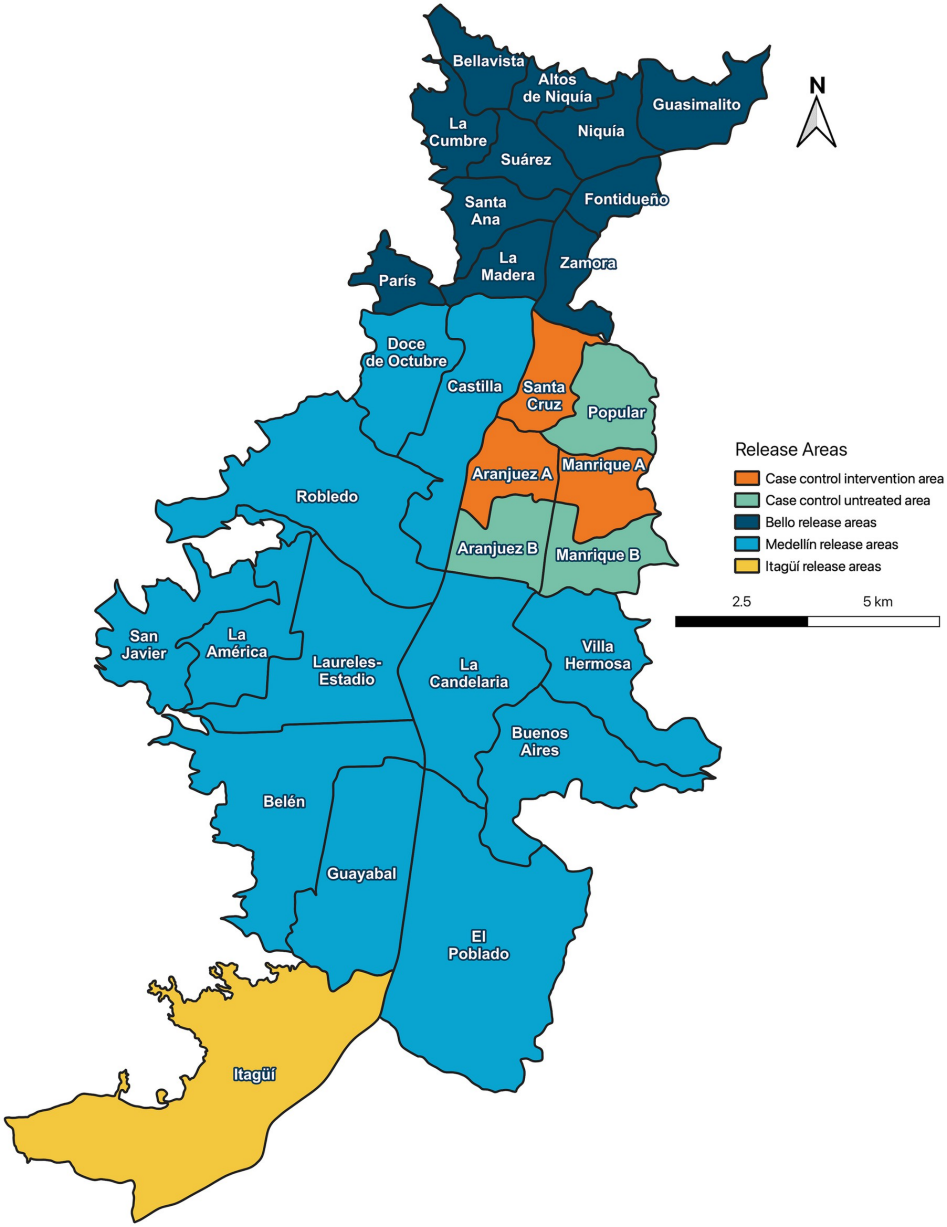
**Deployment of *w*Mel across Medellín, Bello and Itagüí**—combining pragmatic staged (Bello and Medellín) and municipality-wide (Itagüí) deployments (dark blue, blue and yellow) with a case-control study with test-negative design in a focused study area of intervention (orange) and untreated (green) areas (produced in QGIS version 3.28.3 using administrative boundaries freely available from the municipal governments of Bello (https://www.datos.gov.co/Ordenamiento-Territorial/Divisi-n-Pol-tico-Administrativa-Barrios-Bello-Ant/pnhh-ccwd), Medellín (https://www.medellin.gov.co/geomedellin/datosAbiertos/1043), and Itagüí (https://www.datos.gov.co/Ordenamiento-Territorial/Localizaci-n-Geogr-fica-de-los-Barrios-del-Municip/didi-drqa)).

The Aburrá Valley is located in the Department of Antioquia (Fig 1), in the northwest of Colombia, and is among the most populous areas in Colombia. Medellín, Bello and Itagüí are the main urban centres of the valley with populations in 2021 of 2.53 million, 545 thousand and 270 thousand, respectively (3.3 million combined population in an area of 135 km^2^) [24]. The three cities accounted for 91% of the dengue case burden in Antioquia in the ten years 2008–2017 [25].

### Summary of wMel implementation

A detailed description of the implementation of the *w*Mel method within the Aburrá Valley is reported in a concurrent publication [23]. Briefly, after initial pilot releases in the París comuna of Bello between June 2015 and August 2016, staged city-wide deployments of *w*Mel-infected *Ae*. *aegypti* throughout all 10 comunas of Bello commenced in October 2016 and concluded in April 2019 (S1 Fig). Two parallel streams of *w*Mel deployments were undertaken in Medellín: deployments into the intervention arm of a case-control study in one quadrant of Medellín (4 comunas divided into 3 intervention and 3 control areas; August 2017—May 2019) and staged deployments throughout the rest of Medellín (12 comunas; October 2017—October 2019). Subsequent to the completion of participant enrolment for the case-control study in December 2021 (see below), between January and April 2022 *w*Mel-infected mosquitoes were released into the remaining three areas that had served as control areas for the duration of the case-control study. *w*Mel was deployed simultaneously across the whole of Itagüí between August 2019 and December 2020.

*w*Mel introgression into *Ae*. *aegypti* populations throughout release areas was determined through regular monitoring [23] and is summarised in S1 Fig. Mosquitoes were captured through a network of BG-Sentinel traps (BioGents) deployed throughout release areas or via aspirators operated by study personnel. Mosquito *w*Mel infection status was determined by screening with polymerase chain reaction (PCR)-based assays as described elsewhere [23], and *w*Mel prevalence was calculated as the percentage of *Ae*. *aegypti* screened that were *w*Mel-positive, aggregate by comuna and month. The COVID-19 pandemic and associated restrictions disrupted both *w*Mel monitoring and deployment between March 2020 and March 2021, resulting in incomplete monitoring data for most release areas as well as delays in *w*Mel deployment into some areas.

### Quasi experimental study

#### Routine disease surveillance data

The monthly count of notified dengue cases by comuna was obtained from the Health Secretariats of Medellín and Bello, and municipality-level data for all Colombian cities was obtained from the National Health Institute’s National Public Health Surveillance System (Instituto Nacional de Salud [INS] SIVIGILA [25]). Comuna-level data was available from 2009 for Medellín and 2010 for Bello, up to June 2023. Municipality-level data was available from 2008 for all Colombian cities, up to 27 May 2023 (epidemiological week 21). The INS weekly epidemiological bulletins in published in 2023 refer to there being 57 dengue-endemic cities in Colombia with population greater than 100,000 inhabitants [26]. A list of these 57 cities (including Medellín, Bello and Itagüí) was compiled (S1 Table) based on information from the INS weekly epidemiological bulletins [26], census population data [24], and municipality-level dengue notifications data [25].

Nationally, the case definition used for dengue surveillance and reporting includes all laboratory-confirmed dengue cases and any clinically suspected dengue cases who were not laboratory-tested [27]. Suspected dengue cases who test negative for dengue IgM antibody are excluded from national reporting, although these cases are retained in the datasets maintained by the Medellín and Bello Health Secretariats. For consistency with the national datasets, we employed the same case definition and excluded test-negative suspected dengue cases from the comuna-level datasets used in interrupted time series (ITS) analysis for determining the impact of *w*Mel deployments on the incidence of notified dengue. For the ITS analysis we collated dengue case counts aggregated by month and by patients’ comuna of residence (Medellín and Bello) or municipality of residence (Itagüí) to align with the geographical areas used for *w*Mel releases and monitoring. For descriptive analyses of the baseline dengue epidemiology prior to large-scale *Wolbachia* deployments, we used the INS SIVIGILA datasets for all three cities.

Data on notified cases of Zika and chikungunya were also available from the Health Secretariats of Medellín and Bello from 2014–2022, and were analysed as a secondary endpoint.

Population data stratified by age and sex for each municipality was obtained from the Colombian National Administrative Department of Statistics 2018 census [24], and population data by comuna for Bello and Medellín was obtained from the municipal governments.

#### Statistical analysis

Per capital annual dengue incidence (notified cases per 100,000 inhabitants) was calculated for each of the 57 dengue-endemic cities in Colombia, 2008 to 2023. For 2023, data was only available to epidemiological week 21 (27 May 2023), so the calculation of 2023 per capita incidence was annualised by adjusting by a factor of 52/21 = 2.476. For each year 2008–2023, the median and interquartile range of dengue incidence among the 57 dengue-endemic cities was calculated.

The analysis of the public health outcomes of *w*Mel deployments included dengue notification data from January 2008 (Itagüí), January 2009 (Medellín) or January 2010 (Bello) until June 2023. The comuna of París in Bello was excluded from analysis because the pilot releases in 2015 introduced *w*Mel in only one neighbourhood of the comuna, whereas the dengue data was reported for the whole of París comuna and cannot be disaggregated by neighbourhood to consider the pilot release areas separately from the rest of the comuna.

*w*Mel prevalence in months where no *w*Mel monitoring was conducted was interpolated using a regression line between the last *w*Mel monitoring event and the next. The final measured *w*Mel prevalence was carried forward through subsequent months until June 2023. The *w*Mel exposure status of each comuna in each month was defined as untreated (prior to *w*Mel releases), partially treated (*w*Mel releases ongoing or completed but not stably at ≥60% *w*Mel), or fully treated (releases completed and stably at ≥60% *w*Mel). The ‘interruption’ in the ITS analysis is thus represented by the time point at which an individual comuna becomes partially- or fully-treated. An alternative *w*Mel exposure definition used categorised levels of comuna-level (city-level for Itagüí) monthly *w*Mel prevalence as a predictor of dengue incidence (<20%; 20% to <40%; 40% to <60%; ≥60%). Here, comunas can move across *w*Mel levels from month to month and thus each *w*Mel category level may represent a different set of comunas each month.

In Bello and Medellín where data was disaggregated by comuna, the ITS analysis was implemented using mixed-effect negative binomial regression to model the monthly count of dengue case notifications in each comuna as a function of *w*Mel treatment status (fully, partially or untreated in the primary analysis, and by level of *w*Mel prevalence in the secondary analysis), with an offset for population size, calendar month as a fixed-effect covariate, and comuna modelled as a random effect. In Itagüí where data was aggregated at the level of the municipality, a fixed effect negative binomial model was used with an offset for population size and calendar month as a covariate. The models estimate the *w*Mel intervention effect as the dengue incidence rate ratio (IRR) in fully- or partially-treated vs untreated periods (or by stratum of *w*Mel prevalence compared with the lowest stratum), adjusted for seasonality. Robust standard errors were used in all analyses by specifying the vce(cluster *comuna*) option in Stata to account for non-independence of observations within comunas [27].

### Case control study

#### Study design

This clinic-based prospective case-control study used a test-negative design and was designed to measure experimentally the degree to which dengue incidence was reduced in three neighbourhoods where *w*Mel *Wolbachia* had been released (population 323,000 in 6.9 km^2^), compared to three adjacent untreated neighbourhoods (population 331,000 in 8.3 km^2^) (Table 1). Randomised allocation was not feasible due to the small number of clusters, which was driven by the imperative at the time of project conception to prioritise rapid phased deployment throughout the rest of Medellín and Bello, to address an urgent need for novel scalable strategies to address the threat of Zika [22]. Instead, the allocation of the six areas into two arms was done in a way that maximised balance between the arms with respect to measured factors that may be associated with baseline dengue risk.

**Table 1:** Baseline characteristics and release summary by area in the case-control study area.

Area	Study arm	Population(2021)	Total area km^2^	Release area km^2^	# Release rounds	Release start and end dates	Estimated mosquitoes released
Aranjuez A	Intervention	105,873	2.32	2.12	46	23/08/17–15/05/19	3,121,008
Manrique A	Intervention	87,984	2.42	1.89	40	23/08/17–29/03/19	2,426,102
Santa Cruz	Intervention	129,417	2.19	2.08	40	23/08/17–31/03/19	3,132,843
Aranjuez B	Untreated	82,021	2.56	–	–	–	–
Manrique B	Untreated	97,347	2.68	–	–	–	–
Popular	Untreated	151,283	3.10	–	–	–	–

#### Participant recruitment and data collection

To measure the epidemiological endpoint, participants were recruited from a network of 11 clinics across the study area. Febrile patients were invited to participate in the study if they met the following inclusion criteria: fever with a date of onset between 1–4 days prior to the day of presentation to the health care facility; aged ≥3 years old; and lived in the case-control study area for the 10 days preceding illness onset. Participants were not eligible if localizing features suggestive of a specific diagnosis were identified or if they had been enrolled in the previous four weeks. After obtaining written informed consent, basic demographic details, eligibility against the inclusion criteria, illness onset date, and a retrospective travel history encompassing days 3–10 prior to illness onset were recorded in a standardised electronic data collection form. A single 6 ml venous blood sample was collected from all consenting participants on the day of enrolment. Only participants enrolled from the 16th of May 2019, following completion of *w*Mel releases in the intervention area, were included in the analysis dataset. Enrolment was paused between 1 April 2020 and 31 January 2021 due to COVID-19 pandemic restrictions, and concluded on the 31st of December 2021.

#### Case and control classification

The ZDC Multiplex RT-qPCR (Bio-Rad) was used to detect DENV, CHIK and Zika viruses in plasma samples from all enrolled participants. Samples were tested for the presence of dengue NS1 antigen by ELISA (Panbio Dengue Early ELISA [n = 720 samples] or BioRad Platelia [n = 92 samples]), according to manufacturers’ instructions. Samples which tested negative by DENV/CHIK/Zika RT-qPCR and DENV NS1 were tested for dengue IgM antibodies by IgM capture ELISA (Panbio [n = 763 samples] or InBios [n = 46 samples]). Samples positive for DENV in the triplex RT-qPCR were tested in a serotype-specific RT-qPCR to determine the infecting serotype, as described previously [28].

Virologically-confirmed dengue (VCD) cases (primary endpoint) were defined as participants with a positive result in DENV RT-qPCR or NS1 ELISA (S2 Fig). Presumptive dengue cases (secondary endpoint) were defined as participants who tested negative for dengue by DENV RT-qPCR and NS1 ELISA but who had a positive DENV IgM test. Controls were defined as participants meeting the clinical criteria for enrolment, but with negative test results for DENV RT-qPCR, DENV NS1 ELISA, DENV IgM ELISA, CHIK RT-qPCR, and Zika RT-qPCR. Equivocal test results were re-tested and participants with two equivocal test results were excluded from analysis.

#### Sample size calculation

Initial sample size estimates calculated that 88 test-positive cases plus four times as many controls would be sufficient to detect a 50% reduction in dengue incidence with 80% power, based on standard formulae for calculating sample size/power in a case control study (http://www.openepi.com/SampleSize/SSCC.htm). This aligned with the proposed approach to estimating the intervention effect, which compares the exposure odds among test-positive cases versus test-negative controls, with the null hypothesis that the odds of residence in the *Wolbachia* intervention arm is the same among test-positive cases as test-negative controls, and did not account for clustering of participants in the 3 treated and 3 untreated zones. A re-evaluation of sample size requirements was conducted in April 2021 following 14 months of participant enrolment (May 2019 –March 2020 and February–April 2021), to consider the minimum effect size that would be detectable for a smaller sample size than originally estimated, given the 77% efficacy that had since been reported from a cluster randomised trial of *w*Mel in Yogyakarta [14] and the low incidence of dengue in Medellín since case-control enrolment began. This found that 42 test-positive dengue cases (and four times as many controls) would be sufficient to detect a 65% reduction in dengue. Clustering was not explicitly accounted for in the sample size calculation, rather an inflation factor of 1.5 was applied, to give a revised target sample size of 63 virologically-confirmed dengue cases and at least 252 test-negative controls.

#### Statistical analysis

The statistical analysis plan was published [29] and is available in the supporting information (S1 Appendix). The dataset for analysis included all enrolled virologically-confirmed dengue cases and presumptive dengue cases, and all test-negative controls that were matched to a case by calendar quarter of enrolment (S3 Fig). The intention-to-treat analysis considered *w*Mel exposure as a binary classification based on residence in the intervention or untreated area. The intervention effect was estimated from an aggregate odds ratio (OR) comparing the exposure odds (residence in the intervention area) among test-positive cases versus test-negative controls (for data aggregated across all three release areas within each study arm) with cluster-robust variance estimates. Area-level averages for age and sex were included as covariates in the model. The OR provides an unbiased estimate of the relative risk of dengue in *Wolbachia*-treated versus untreated areas, providing that the key assumptions underlying a test-negative design are upheld, namely that test-negative controls are allowed to include participants who may test positive for dengue at any other time during the study period, and the distribution of non-dengue febrile illness is not associated with the intervention status [30,31]. Efficacy of the intervention was calculated as 100*(1-aggregate OR).

The per-protocol analysis considered *w*Mel exposure as a quantitative index based on measured *w*Mel prevalence in local *Ae*. *aegypti* mosquitoes in the participant’s area of residence, and in locations visited by the participant during the period 3–10 days prior to illness onset. A weighted *Wolbachia* exposure index (WEI) was defined for each participant, as follows: the aggregate *w*Mel prevalence for each of the six areas was calculated each month as the number of *w*Mel-positive *Ae*. *aegypti* mosquitoes in that area divided by the total number of *Ae*. *aegypti* that were screened for *w*Mel in that area. The WEI for each participant was then calculated by multiplying the area-level *w*Mel prevalence (in the calendar month of participant enrolment) at each of the locations visited, by the proportion of time spent at each location, and summing across locations to give a value on a continuous scale from 0 to 1. An additional per-protocol analysis was conducted in which the WEI was calculated using only the area-level *w*Mel prevalence in the participant’s area of residence (in the calendar month of participant enrolment), ignoring the participant’s recent travel history. Cases and controls were classified by strata of their WEI: 0–0.2; 0.2–0.4; 0.4–0.6; 0.6–0.8; and 0.8–1. A mixed-effects logistic regression was used to model the relationship between WEI and dengue incidence, with age and sex as covariates, and area as a random intercept term. The WEI strata were modelled as an unordered covariate to calculate stratum-specific ORs (relative to the baseline 0–0.2 stratum). Efficacy was calculated as 100*(1-OR).

## Results

### Baseline dengue epidemiology

In the ten years from January 2008 to December 2017 prior to city-wide *w*Mel releases in Bello, Medellín, and Itagüí, there were 60,896 dengue cases notified to the national surveillance system from the three cities (Bello: 5569 cases corresponding to 378 cases per 1000 population; Medellín: 47,212 cases, 20 per 1000; Itagüí: 8115 cases, 777 per 1000). Forty-five percent (n = 27,166) were classified as either laboratory-confirmed or epidemiologically-linked cases, and the remainder as probable cases. Less than one percent of notified cases were classified as severe dengue (n = 539) and 21.3% of cases (n = 12,959) were hospitalised. The median age of dengue cases was 28 years (interquartile range 16–44 years), and was similar among cities, between severe and non-severe cases and between hospitalised and non-hospitalised cases. Overall, 51.4% of dengue cases were female but after accounting for the demographic structure of the underlying population, dengue incidence was slightly higher in males (20.1 cases per 1000 males during 10 years vs 19.0 cases per 1000 females). S4 Fig shows the age and sex distribution of dengue cases notified from the three cities between 2008 and 2017.

### Public health outcomes of wMel deployments: Quasi experimental study

Among 10 comunas in Bello, *w*Mel prevalence was stably ≥60% (i.e. fully treated) immediately following the end of releases in two comunas, and within 4–21 months (median 9.5 months) post-release in the remaining eight. Of the 18 release areas in Medellín, *w*Mel prevalence was stably ≥60% within 2 months of the end of releases in five areas, and within 4–17 months (median 8 months) in another five areas. Eight release areas remained partially treated (not stably at ≥60% *w*Mel) at the time of the last entomological monitoring between July 2021 and January 2022, 21–34 months after the end of releases. In Itagüí, *w*Mel prevalence was stably ≥60% (i.e. fully-treated) immediately following the end of releases.

**Fig 2:**
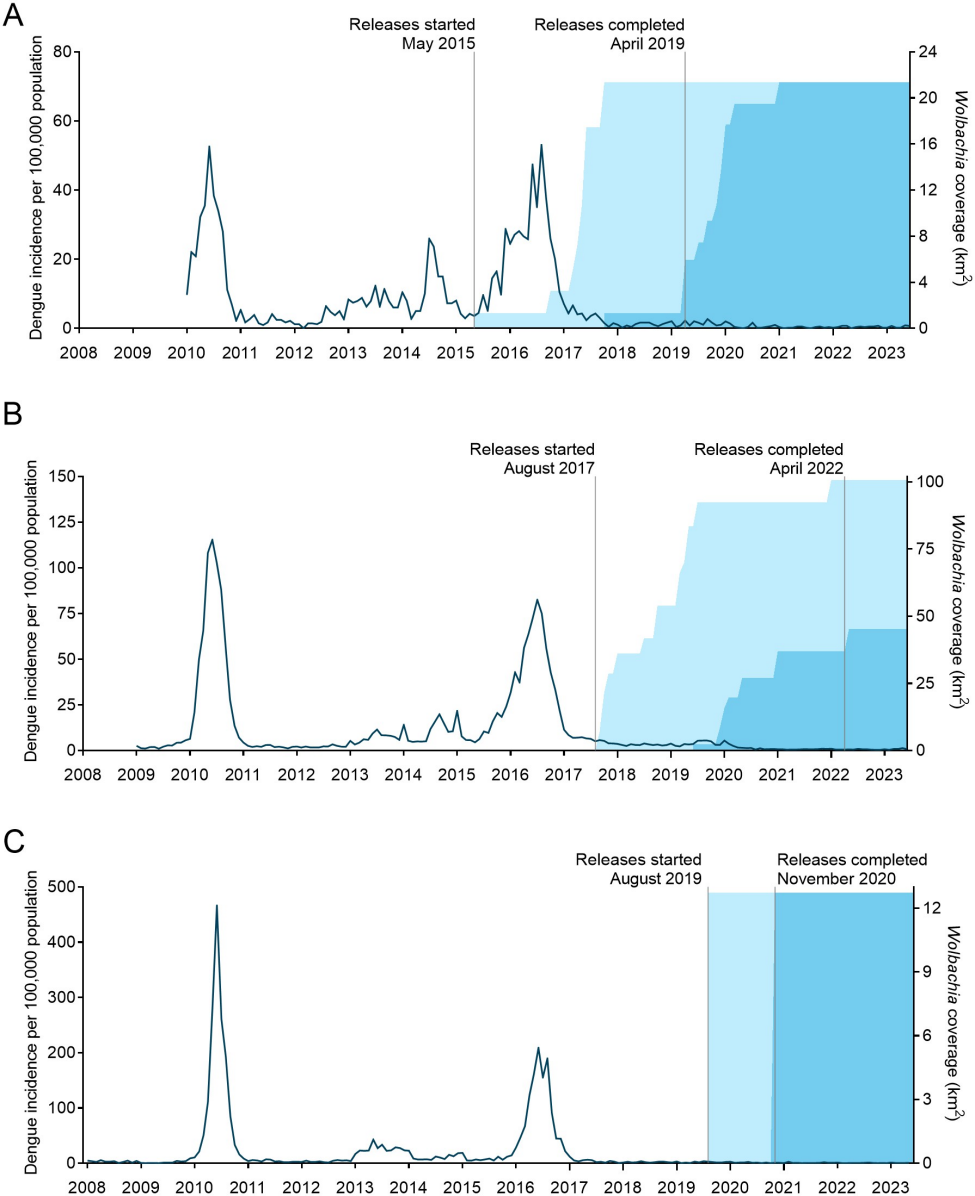
Dengue incidence in *w*Mel-release areas in A) Bello, B) Medellín and C) Itagüí. Dark blue line is the monthly incidence of dengue case notifications per 100,000 population (left-hand Y axis; note different scale among graphs) from January 2008 (Itagüí)/2009 (Medellín)/2010 (Bello) to June 2023. Light blue shading indicates the *w*Mel area coverage (km^2^) in partially *w*Mel-treated areas (*w*Mel releases were ongoing or completed but not stably at ≥60% *w*Mel), and darker blue shading indicates the *w*Mel area coverage (km^2^) in fully *w*Mel-treated areas (releases were completed and stably at ≥60% *w*Mel) (right-hand Y axis).

**Fig 3:**
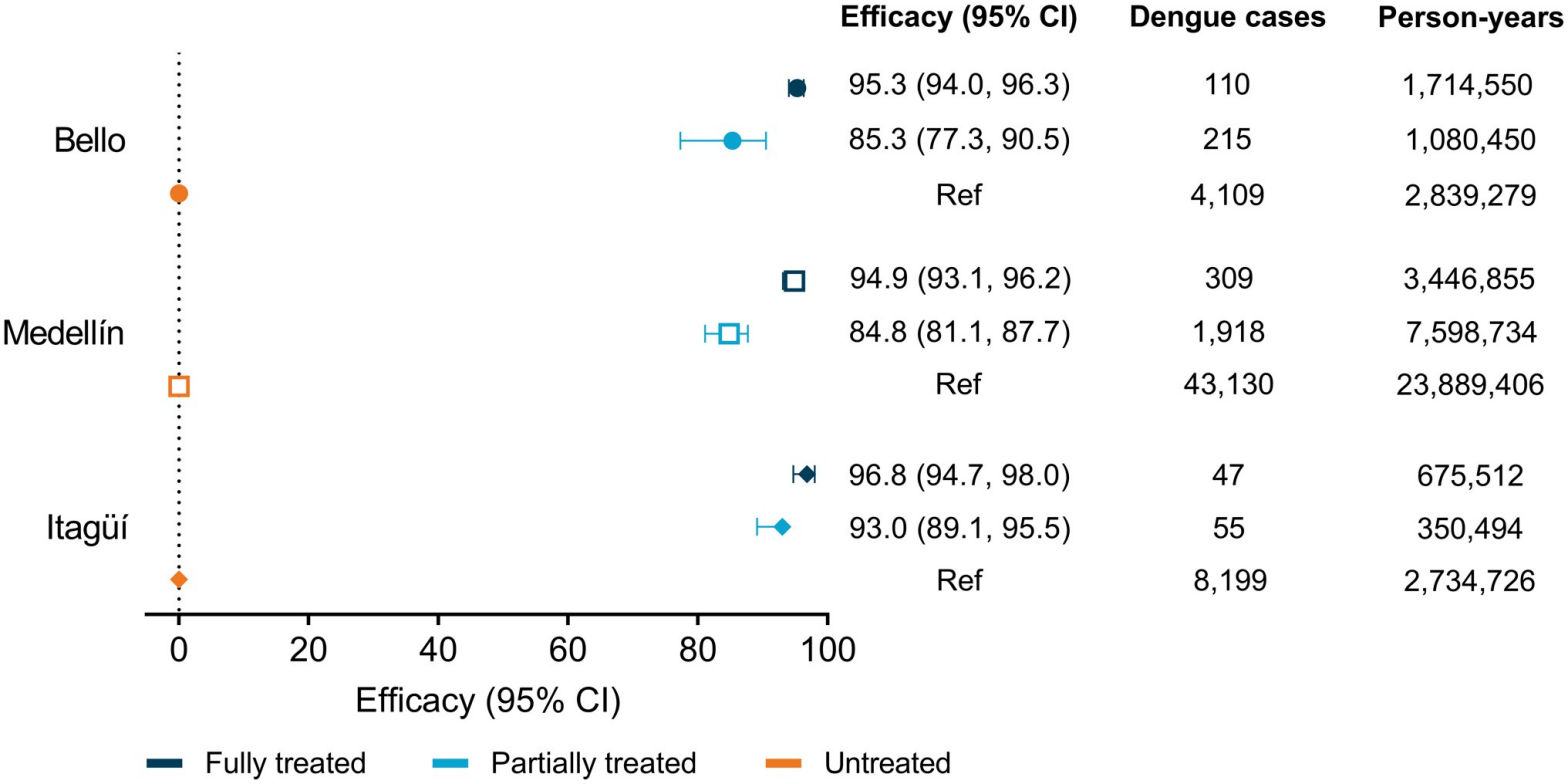
Efficacy of the *w*Mel intervention against suspected dengue notified to the routine disease surveillance system in the partially or fully *w*Mel-treated period compared to the untreated period. Point estimates (symbols) and 95% confidence intervals (horizontal bars) from interrupted time series analysis of monthly dengue case notifications to the routine surveillance system. Efficacy is expressed as 100x(1-IRR). *w*Mel exposure was defined as ‘untreated’ prior to *w*Mel releases, ‘partially treated’ if *w*Mel releases were ongoing or completed but not stably at ≥60% *w*Mel, and ‘fully treated’ if releases were completed and stably at ≥60% *w*Mel.

The incidence of notified dengue in Bello, Medellín and Itagüí before, during and after *w*Mel deployments is shown in Fig 2. Stable introgression of *w*Mel into local *Ae*. *aegypti* populations was associated with a significant reduction in dengue incidence in each municipality. In Bello, there were 110 dengue cases (6.4 per 100,000 person-years) notified in the fully treated period and 215 cases (19.9 per 100,000 person-years) notified in the partially treated period, compared to 4,109 cases (144.7 per 100,000 person-years) in the untreated period (Fig 3). In the interrupted time series analysis, this was equivalent to a 95% reduction in dengue incidence (incidence rate ratio 0.047 [95% CI 0.037, 0.060]) in the fully treated period and an 85% reduction (IRR 0.147 [95% CI 0.095, 0.227]) in the partially treated period compared to the untreated period. In Medellín, there were 309 cases (9.0 per 100,000 person-years) notified in the fully treated period and 1,918 (25.2 per 100,000 person-years) in the partially treated period, compared to 43,130 cases (180.5 per 100,000 person-years) in the untreated period (Fig 3), equating to a 95% (IRR 0.051 [95% CI 0.038, 0.069]) and an 85% (IRR 0.152 [95% CI 0.123, 0.189]) reduction in dengue, respectively. In Itagüí, there were 47 cases (7.0 per 100,000 person-years), 55 cases (15.7 per 100,000 person-years) and 8,199 cases (299.8 per 100,000 person-years) notified in the fully treated, partially treated and untreated periods respectively, equating to a 97% (IRR 0.032 [95% CI 0.020, 0.053]) and a 93% (IRR 0.070 [95% CI 0.045, 0.110]) reduction in dengue.

**Fig 4:**
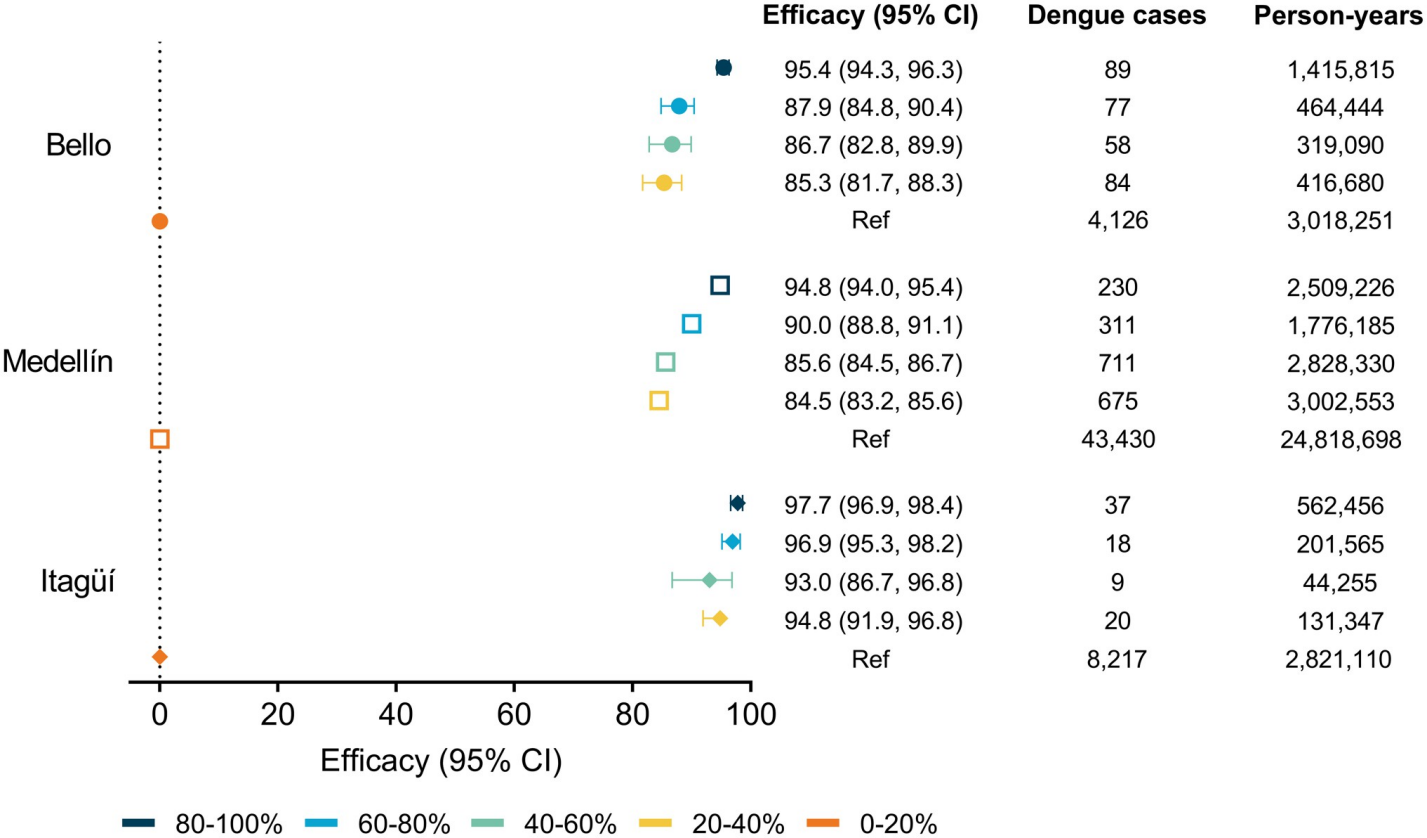
Efficacy of the *w*Mel intervention against suspected dengue notified to the routine disease surveillance system by categorised level of commune-level (Bello and Medellín) or city-level (Itagüí) *w*Mel prevalence. Point estimates (symbols) and 95% confidence intervals (horizontal bars) from interrupted time series analysis of monthly dengue case notifications to the routine surveillance system. Efficacy is expressed as 100x(1-IRR).

Significant reductions in dengue incidence were observed for all category levels of *w*Mel prevalence, with no apparent dose response relationship (Fig 4). In Itagüí, reductions in dengue of similar magnitudes ranging from 93–98% were seen across all *w*Mel levels compared to the lowest category level. In Bello and Medellín, dengue incidence was 95% lower, when comparing the highest *w*Mel level with the lowest, and significant reductions in dengue incidence (range 85–90%) were also observed at intermediate levels of *w*Mel.

In Bello, there were 106 chikungunya cases notified between 2014–2019, only one of which (in December 2019) was resident in a fully-treated area (*w*Mel prevalence ≥60%), and 52 Zika cases notified between 2015–2017, all from untreated areas. There have been no chikungunya or Zika cases notified from Bello since December 2019 and June 2017, respectively. In Medellín there were 794 chikungunya cases notified between 2014–2020 and 494 Zika cases notified between 2015–2022, none of which were resident in a fully-treated area.

### Notified dengue incidence in wMel-treated versus untreated Colombian cities, pre- and post-intervention

**Fig 5:**
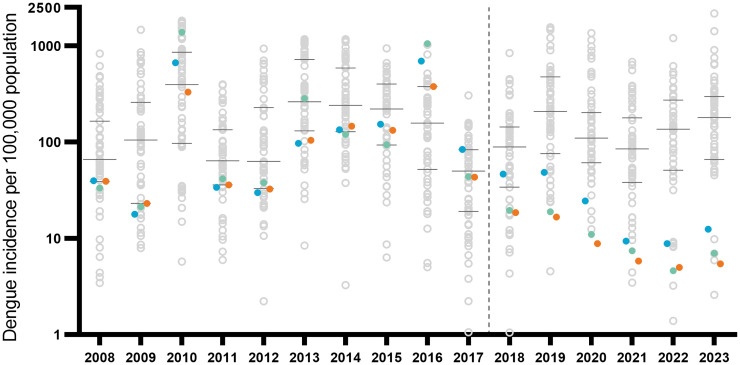
Annualised dengue incidence per 100,000 population in 57 dengue-endemic Colombian cities with population >100,000, 2008 to June 2023. Coloured filled circles indicate annual dengue incidence in the three cities in the Aburrá Valley where staged roll-out of the *w*Mel intervention was implemented between 2017 and 2021: Bello (orange), Medellín (blue) and Itagüí (green). Open grey circles represent annual dengue incidence in the remaining 54 cities (note the log10 scale on the Y-axis). The horizontal lines indicate the median and interquartile range of dengue incidence among the 57 cities in each year. The vertical dashed line roughly demarcates the pre-intervention and post-intervention periods.

Among the 57 cities in Colombia with population >100,000 inhabitants and classified as dengue-endemic (S1 Table), there was substantial heterogeneity in per capita dengue incidence both within and between years, 2008–2023 (Fig 5). All three *w*Mel-treated cities in the Aburrá Valley have ranked among the 10 lowest-incidence cities every year since 2019, whereas this was not the case in any year 2010–2017, prior to *Wolbachia* deployments.

### Public health outcomes of wMel deployments: Case-control study

#### Participant characteristics

Among 25,304 febrile patients presenting to the participating 11 primary care clinics between 10 November 2017 and 31 December 2021 and screened for eligibility, 1,665 were found to be eligible and consented to be enrolled. A total of 725 participants (351 from the intervention area and 374 from the untreated area) met the criteria for inclusion in the analysis dataset (S3 Fig). The most common reason for exclusion from analysis was enrolment prior to *w*Mel establishment (88% of excluded participants). Within the analysis dataset, the median (interquartile range) age was 23.8 years (9.2–35.9 years) among participants from the intervention area and 24.5 years (11.0–39.2 years) among participants from the untreated area, and 49% and 53% of participants were female, respectively.

Twenty-three participants were classified as VCD on the basis of either a positive DENV PCR result (n = 20) or a positive NS1 antigen result with negative PCR (n = 3). The infecting serotype was DENV1 in 17 participants, DENV3 in two participants, and could not be determined for the remaining four VCDs. No DENV2 or DENV4 cases were detected in this study, nor any Zika or chikungunya cases. An additional 15 participants were negative in DENV PCR and NS1 antigen testing but were DENV IgM-positive and were classified as presumptive dengue cases, giving a total of 38 participants meeting the secondary endpoint of virologically-confirmed or presumptive dengue (i.e. any dengue).

#### Dengue incidence in the intervention vs untreated area (intention-to-treat analysis)

**Fig 6:**
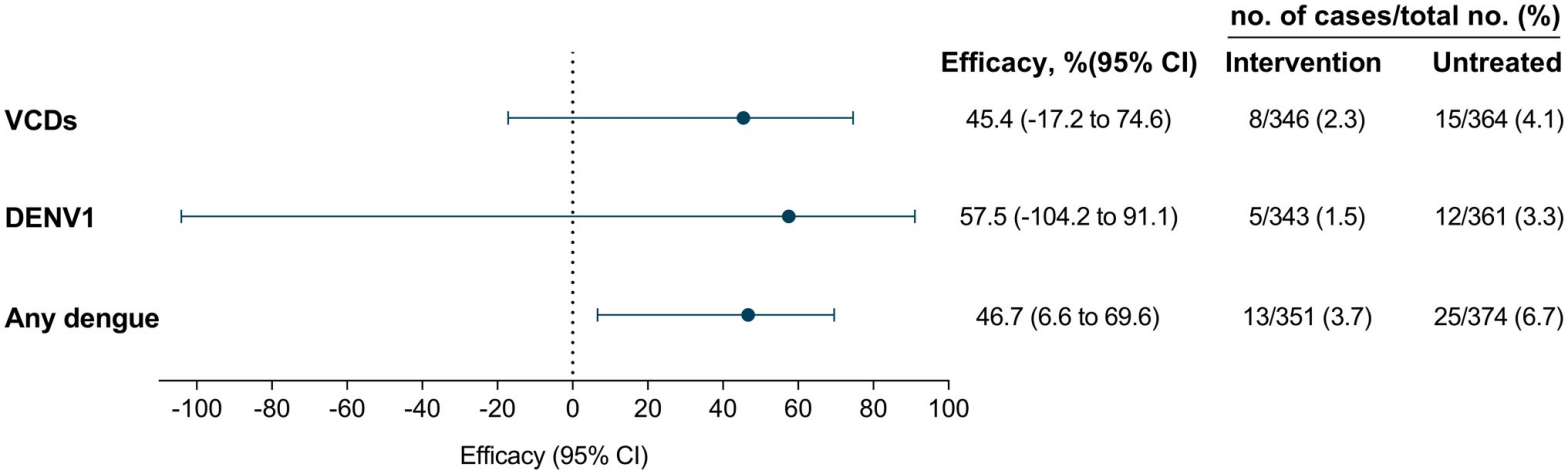
Efficacy in the case-control study intention-to-treat analysis. Shown is the protective efficacy (expressed as 100×(1−OR)) of *w*Mel-infected *Aedes aegypti* deployments against virologically-confirmed dengue of any serotype (VCD), serotype 1 dengue (DENV1), and VCD and presumptive dengue (any dengue).

The incidence of VCDs was 45% lower in the intervention area compared to the untreated area, though this was not statistically significant (8/23 VCDs and 338/687 test-negatives resident in intervention and 15/23 VCDs and 349/687 test-negatives in untreated areas; OR 0.55 [95% CI 0.25, 1.17]) (Fig 6). A similar but statistically significant reduction was observed for the endpoint of any dengue, including both VCDs and presumptive dengue cases (13/38 dengue cases in intervention and 25/38 in untreated areas; OR 0.53 [95% CI 0.30, 0.93]). Serotype-specific analysis could only be performed for DENV1 cases, which showed a non-significant 57% reduction in DENV1 cases in the intervention compared to the untreated area (OR 0.43 [95% CI 0.09, 2.04]).

#### Dengue incidence across Wolbachia Exposure Index strata (per-protocol analysis)

**Fig 7:**
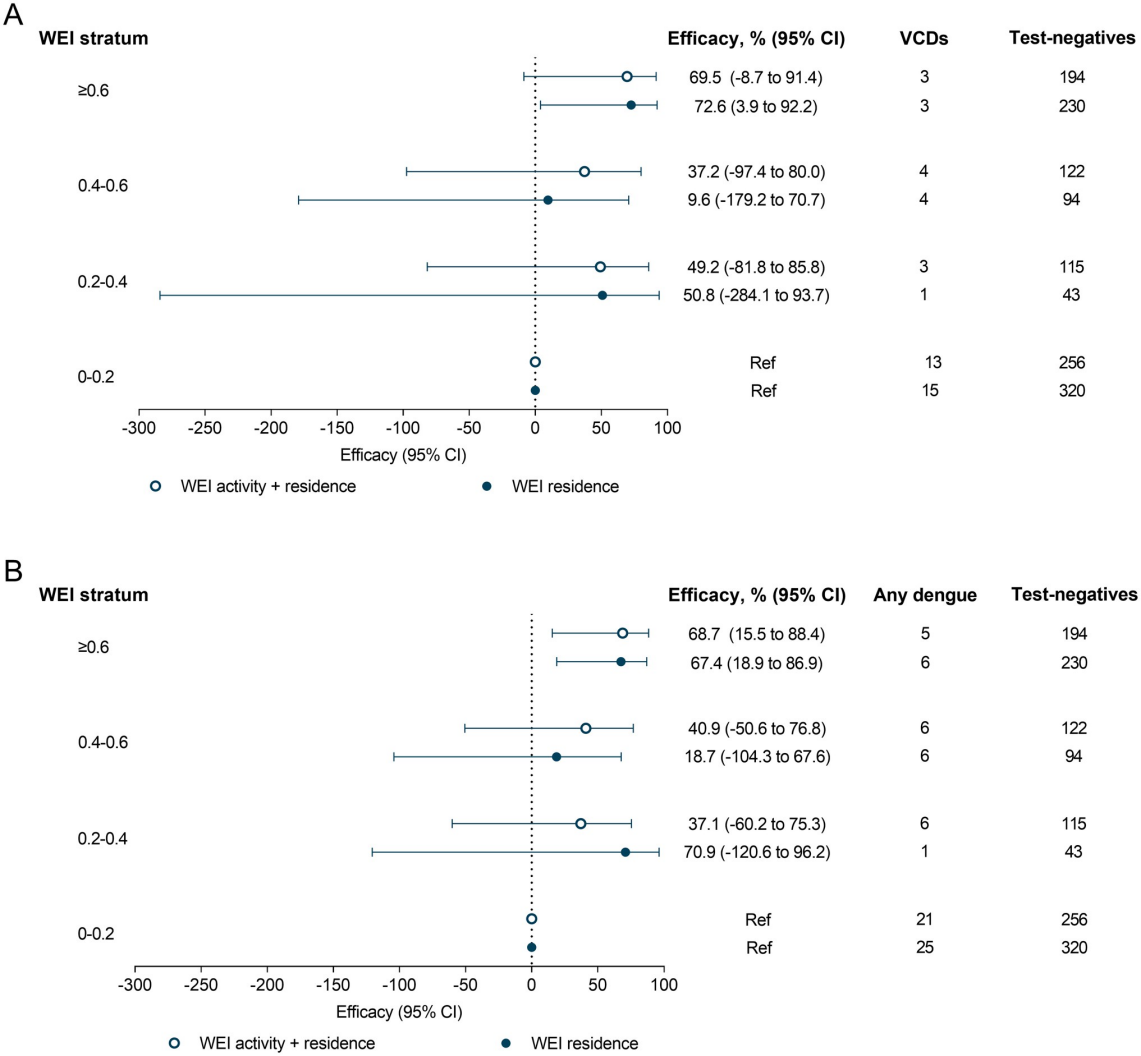
Percentage efficacy of the *w*Mel intervention against (A) virologically-confirmed dengue and (B) VCDs and presumptive dengue cases (any dengue) according to *Wolbachia* exposure index. Markers show stratum-specific efficacy (and 95% confidence intervals) against dengue by categorised level of *Wolbachia* exposure index, with WEI based on duration-weighted *w*Mel frequencies in the cluster of residence and other visited locations (open circles) or *w*Mel frequency in cluster of residence only (closed circles).

After tabulation of VCDs by WEI stratum, the decision was made to combine the top 2 strata to boost sample size. The incidence of VCD was 73% lower (95% CI 4%, 92%) among participants in the top WEI stratum (≥0.6) compared to the lowest stratum (0–0.2) for WEI calculated based on duration-weighted *w*Mel frequencies in the cluster of residence only, and 10% and 51% lower among those with WEI 0.4–0.6 and 0.2–0.4, though these differences were not statistically significant (Fig 7A). Similar results were found for WEI calculated based on duration-weighted *w*Mel frequencies in the cluster of residence and other visited locations, though none of these estimates reached statistical significance. There was no indication of a threshold effect nor a dose response relationship with increasing WEI, though we acknowledge that the number of cases in each WEI stratum above 0.2 was very small (range 1–4). The incidence of any dengue was 69% (95% CI 16, 88%) and 67% (95% CI 19, 87%) lower in participants with WEI ≥0.6 compared to the lowest stratum, for WEI calculated based on residence and visited locations and WEI calculated based on residence alone, respectively (Fig 7B).

## Discussion

Since the large-scale roll-out of *Wolbachia*-infected *Ae*. *aegypti* mosquitoes across a continuous population of 3.3 million people in Bello, Medellín and Itagüí, Colombia, the incidence of notified dengue cases has been 95–97% lower than during the decade prior to *Wolbachia* introduction, after adjustment for seasonal trends. A causal association between the *Wolbachia* deployments and reduced dengue incidence is supported by the results of a parallel prospective case-control study, which found that the incidence of virologically-confirmed and presumptive dengue cases detected prospectively in outpatient clinics was significantly lower in three *Wolbachia*-treated neighbourhoods in northeast Medellín than in three well-matched untreated neighbourhoods. The infecting serotype was DENV1 in three-quarters of cases detected in the case-control study.

The reduction in notified dengue incidence following *w*Mel *Wolbachia* establishment in Bello, Medellín and Itagüí is consistent with results from Australia [11,12], Indonesia [13,14], and Brazil [17,19], and with model predictions of a collapse in dengue virus transmission following introduction of *Wolbachia* into local *Ae*. *aegypti* populations [32,33]. The Aburrá Valley, where Bello, Medellín and Itagüí are located, experienced large dengue epidemics in 2010 and 2016, but has seen very little dengue since. The end of the 2016/2017 epidemic coincided with the start of the phased *w*Mel deployments across Medellín and most of Bello, and it is plausible that the post-intervention period has coincided with a natural trough in dengue incidence. However, the average annual incidence since city-wide *w*Mel releases commenced in 2017 has been lower than any other period previously recorded, and the sustained suppression of dengue through seven typical transmission seasons from January 2017 to June 2023 gives increasing confidence in a *Wolbachia*-mediated effect.

Due to the infrequency of dengue epidemics in the Aburrá Valley in the baseline period, and because secular trends and other parallel control measures can confound the demonstration of a *Wolbachia* intervention effect in a before-and-after analysis, we designed *a priori* a prospective case-control study to evaluate experimentally the public health impact of *Wolbachia* releases in one area of Medellín, alongside the time series analysis of routine disease surveillance data for all three cities [22]. Randomised allocation of the *Wolbachia* intervention was not feasible, given the small number of neighbourhoods within the case-control area and the necessarily pragmatic approach to deployments in Medellín in order to achieve large-scale coverage within a short time frame. However, the allocation of the six neighbourhoods into two arms of the case-control study was done in a way to maximise balance between the arm with respect to measured factors that may be associated with baseline dengue risk, in order to minimise risk of bias in the estimation of the *Wolbachia* intervention effect. Although the sustained suppression of dengue throughout Medellín, Bello and Itagüí since enrolment began in 2019 meant that the case-control study was unable to reach its target sample size of 63 enrolled dengue cases in 2.5 years, two-thirds of the 23 virologically-confirmed dengue cases (and an additional 15 presumptive dengue cases) enrolled were resident in the untreated neighbourhoods. The results showed a 47% reduction in the incidence of any dengue (virologically-confirmed or presumptive) among residents of *Wolbachia*-treated neighbourhoods compared to untreated neighbourhoods. Human movement between neighbourhoods, incomplete *Wolbachia* establishment in treated areas, and *Wolbachia* contamination into untreated areas are likely to have limited the measurement of *Wolbachia*’s true effectiveness in the primary analysis of the case-control study. A per-protocol analysis which attempted to account for this exposure misclassification using the measured *Wolbachia* prevalence in the cluster of residence and at other locations visited during the week prior to illness demonstrated a 69% reduction in dengue incidence among participants with highest *Wolbachia* coverage levels, compared to those with the lowest.

The rate of *Wolbachia* establishment in local *Ae*. *aegypti* populations was variable among the Aburrá Valley release areas, with stable *w*Mel prevalence above 60% achieved immediately post-release in Itagüí; in a median of 8 months (range 1–21 months) post-release in Bello; and in a median of 16 months in Medellín (minimum 1 month, with stable establishment not yet verified in 7/18 areas at last monitoring). Sustained suppression of dengue incidence was observed throughout all three cities in the period since *Wolbachia* releases despite this heterogeneity, consistent with the previously published results of field trials in the Brazilian cities of Niteroi and Rio, which showed significant reductions in dengue and chikungunya incidence even with intermediate *w*Mel levels [17,19]. The complexities in achieving homogeneous *Wolbachia* introgression in these large complex urban environments are likely to be common to many other tropical urban centres—including challenges in accessing high-rise areas, gated communities and informal or insecure settlements, diversity in *Ae*. *aegypti* ecology across the city, and spatial heterogeneity in mosquito abundance. Long-term entomological and public health monitoring in the Aburrá Valley will be valuable for ascertaining the degree to which *Wolbachia* prevalence continues to increase without further intervention, as well as for demonstrating the durability of *Wolbachia* once established in the mosquito population and the long-term impact of *Wolbachia* on Aedes-borne disease incidence. Recent evidence from the earliest *w*Mel *Wolbachia* release sites in northern Queensland, Australia, shows *Wolbachia* self-sustaining at a high prevalence 7–10 years post-release with few genomic changes [34,35], and local dengue transmission has been effectively eliminated following area-wide releases [11,12]. In the earliest Colombian release area in París, Bello, *w*Mel prevalence was >90% when last monitored in September 2021, more than five years after the completion of releases [23], supportive of an expectation that the area-wide establishment of *Wolbachia* in *Ae*. *aegypti* mosquito populations throughout the Aburrá Valley will be effective for many years in controlling dengue and other Aedes-borne diseases.

A limitation of relying on routine disease surveillance data to evaluate the long-term public health impact of *Wolbachia* is that case notification in Colombia is based on a clinical diagnosis of dengue, without requiring confirmatory laboratory tests such as NS1 antigen detection or nucleic acid tests for viral RNA. This means that a subset of notified cases will be febrile illness of another aetiology, and these ‘false positive’ dengue cases will continue to be reported even in the absence of true local transmission. Furthermore, the case notification data does not reliably distinguish between autochthonous dengue cases and ‘imported’ cases with a recent travel history outside of the city of residence, so increased dengue activity elsewhere in Colombia could plausibly lead to an uptick in cases resident in the Aburrá Valley release areas but who acquired their infection elsewhere. Nonetheless, routine surveillance data provides a readily available and pragmatic signal for monitoring the long-term effectiveness of *Wolbachia*.

Around half of Colombia’s population live in dengue endemic areas, and the frequency and magnitude of dengue outbreaks is increasing: in six of the ten years 2010–2019, the annual reported dengue cases in Colombia exceeded any of the previous thirty years [36]. Central Latin American countries—including Colombia—are among the regions predicted to see the largest increases in populations affected by increased temperature suitability for dengue transmission in a warming climate [2]. Conventional approaches to dengue control based on chemical control of adult and immature mosquitoes and environmental management to reduce breeding sites, together with effective clinical management, are the mainstay of dengue control programs in endemic countries but have been unable to curtail the spread of dengue. The urgent need for coordinated and sustainable strategies for dengue control is reflected in the World Health Organization’s launch in March 2022 of the Global Arbovirus Initiative [37], one pillar of which is focused on the scale-up and integration of innovative evidence-based interventions for Aedes-borne disease control. In September 2020, *Wolbachia* implementation began in the city of Cali in southwest Colombia, with phased deployments reaching more than 700,000 Cali residents by September 2022 [38]. A recent economic analysis indicates that the initial financial investment required to implement *Wolbachia* in high-burden Colombian cities would generate sustained savings in the long term from the significant reduction in dengue cases and consequent offsets in healthcare and vector control costs [39], consistent with previous findings from Indonesia that *Wolbachia* releases would be highly cost-effective—and even cost-saving—in high-density urban areas [40]. The results presented here demonstrate the feasibility, acceptability and real-world effectiveness of implementing *Wolbachia* across large urban populations and, alongside previously published results, support the reproducibility of this effectiveness across different ecological settings.

## Supporting information

S1 Table List of 57 dengue-endemic municipalities in Colombia with population >100,000 inhabitants, based on information from Colombia National Institute of Health (INS) weekly epidemiological bulletins, in 2023 [26], municipality-level dengue notifications data [25], and national population data [24]. (DOCX)

S1 Fig *w*Mel introgression by commune in Bello and Medellín, and in Itagüí, July 2016 –July 2022. Points indicate the *wM*el infection prevalence in local *Aedes aegypti* mosquito populations categorised into levels. Light blue shading indicates the period during which the area is considered ‘partially treated’, commencing from the beginning of *w*Mel releases. Dark blue shading indicates the period during which the area is considered ‘fully treated’, defined as *w*Mel releases completed and *w*Mel prevalence stably at ≥60%. Absence of shading indicates no *w*Mel releases in that area. Itagüí was not disaggregated by commune as *w*Mel was released simultaneously across the whole city.
(TIF)

S2 Fig Flowchart of data and sample collection procedures and diagnostic algorithm. Blue boxes indicate participant recruitment and enrolment activities undertaken at health clinics, including screening against inclusion/exclusion criteria, obtaining written informed consent, and collection of demographic and travel history data and a blood sample. Yellow boxes indicate the laboratory diagnostic testing performed at the project laboratory, the results of which (white boxes) will be used to classify participants as virologically confirmed dengue, presumptive dengue, Zika or chikungunya cases, or arbovirus-negative controls (grey boxes) according to the algorithm shown.
(TIF)

S3 Fig Participant enrolment and inclusion in the analysis dataset. The commonest reasons for exclusion from the analysis dataset were enrolment before the predefined time point of *w*Mel establishment (16 May 2019) and enrolment in a calendar quarter without any VCD and presumptive dengue cases (‘unmatched controls’; July—September 2021).
(TIF)

S4 Fig Dengue cases notified to the Colombian National Public Health Surveillance System (SIVIGILA) from Bello, Medellin and Itagui between January 2008 and December 2017, by age and sex. Bars show dengue case numbers and lines show per capita incidence in each five-year age band for males (blue) and females (green), aggregated across the three cities and ten years 2008–2017. Data sources: dengue case data (Instituto Nacional de Salud: http://portalsivigila.ins.gov.co/); age- and sex-specifc population by municipality from 2018 census (DANE: https://sitios.dane.gov.co/cnpv/).
(TIF)

S1 Appendix Statistical analysis plan. Published 31 March 2022 at https://www.clinicaltrials.gov/study/NCT03631719.
(PDF)

S1 STROBE Checklist STROBE checklist. Completed checklist of items that should be included in reports of case-control studies.
(DOC)
