# Supplementary material for: Reduced dengue incidence following city-wide wMel Wolbachia mosquito releases throughout three Colombian cities: Interrupted time series analysis and a prospective case-control study
Source: PLoS Negl Trop Dis. 2023 Nov 30;17(11):e0011713. doi: 10.1371/journal.pntd.0011713 (PMC10688673; doi:10.1371/journal.pntd.0011713)
Supplement: S1 Table — (DOCX) [file pntd.0011713.s001.docx]

**S1 Table: List of 57 dengue-endemic municipalities in Colombia with population >100,000 inhabitants**, based on information from Colombia National Institute of Health (INS) weekly epidemiological bulletins, in 2023 [26], municipality-level dengue notifications data [25], and national population data [24].

| **Department** | **Municipality** | **Population (2023)** | **Department** | **Municipality** | **Population (2023)** |
| --- | --- | --- | --- | --- | --- |
| Antioquia | Medellín | 2,653,729 | Magdalena | Santa Marta | 557,388 |
| Antioquia | Apartadó | 133,811 | Magdalena | Ciénaga | 128,647 |
| Antioquia | Bello | 578,376 | Meta | Villavicencio | 558,299 |
| Antioquia | Envigado | 253,699 | Nariño | San Andrés de Tumaco | 257,631 |
| Antioquia | Itagüí | 303,766 | Norte de Santander | San José de Cúcuta | 795,608 |
| Antioquia | Turbo | 136,374 | Norte de Santander | Ocaña | 132,387 |
| Atlántico | Barranquilla | 1,327,209 | Norte de Santander | Villa del Rosario | 113,903 |
| Atlántico | Malambo | 145,396 | Quindio | Armenia | 316,926 |
| Atlántico | Sabanalarga | 104,207 | Risaralda | Pereira | 490,464 |
| Atlántico | Soledad | 692,799 | Risaralda | Dosquebradas | 230,086 |
| Bolívar | Cartagena de Indias | 1,065,570 | Santander | Bucaramanga | 623,378 |
| Bolívar | Magangué | 145,145 | Santander | Barrancabermeja | 216,219 |
| Bolívar | Turbaco | 117,501 | Santander | Floridablanca | 315,981 |
| Caquetá | Florencia | 178,640 | Santander | Girón | 176,418 |
| Cauca | Popayán | 333,382 | Santander | Piedecuesta | 187,763 |
| Cauca | Santander de Quilichao | 115,745 | Sucre | Sincelejo | 304,026 |
| Cesar | Valledupar | 559,462 | Tolima | Ibagué | 545,210 |
| Cesar | Aguachica | 124,553 | Valle del Cauca | Cali | 2,297,230 |
| Córdoba | Montería | 516,217 | Valle del Cauca | Buenaventura | 318,003 |
| Córdoba | Cereté | 110,723 | Valle del Cauca | Guadalajara de Buga | 131,499 |
| Córdoba | Lorica | 117,924 | Valle del Cauca | Cartago | 140,022 |
| Córdoba | Sahagún | 112,585 | Valle del Cauca | Jamundí | 170,492 |
| Cundinamarca | Fusagasugá | 170,039 | Valle del Cauca | Palmira | 361,375 |
| Cundinamarca | Girardot | 121,090 | Valle del Cauca | Tuluá | 223,191 |
| Chocó | Quibdó | 133,906 | Valle del Cauca | Yumbo | 112,272 |
| Huila | Neiva | 373,129 | Arauca | Arauca | 101,457 |
| Huila | Pitalito | 131,735 | Casanare | Yopal | 181,548 |
| La Guajira | Riohacha | 212,314 |  |  |  |
| La Guajira | Maicao | 194,675 |  |  |  |
| La Guajira | Uribia | 199,987 |  |  |  |
