# Supplementary figures and images for: Reduced dengue incidence following city-wide wMel Wolbachia mosquito releases throughout three Colombian cities: Interrupted time series analysis and a prospective case-control study
Source: PLoS Negl Trop Dis. 2023 Nov 30;17(11):e0011713. doi: 10.1371/journal.pntd.0011713 (PMC10688673; doi:10.1371/journal.pntd.0011713)

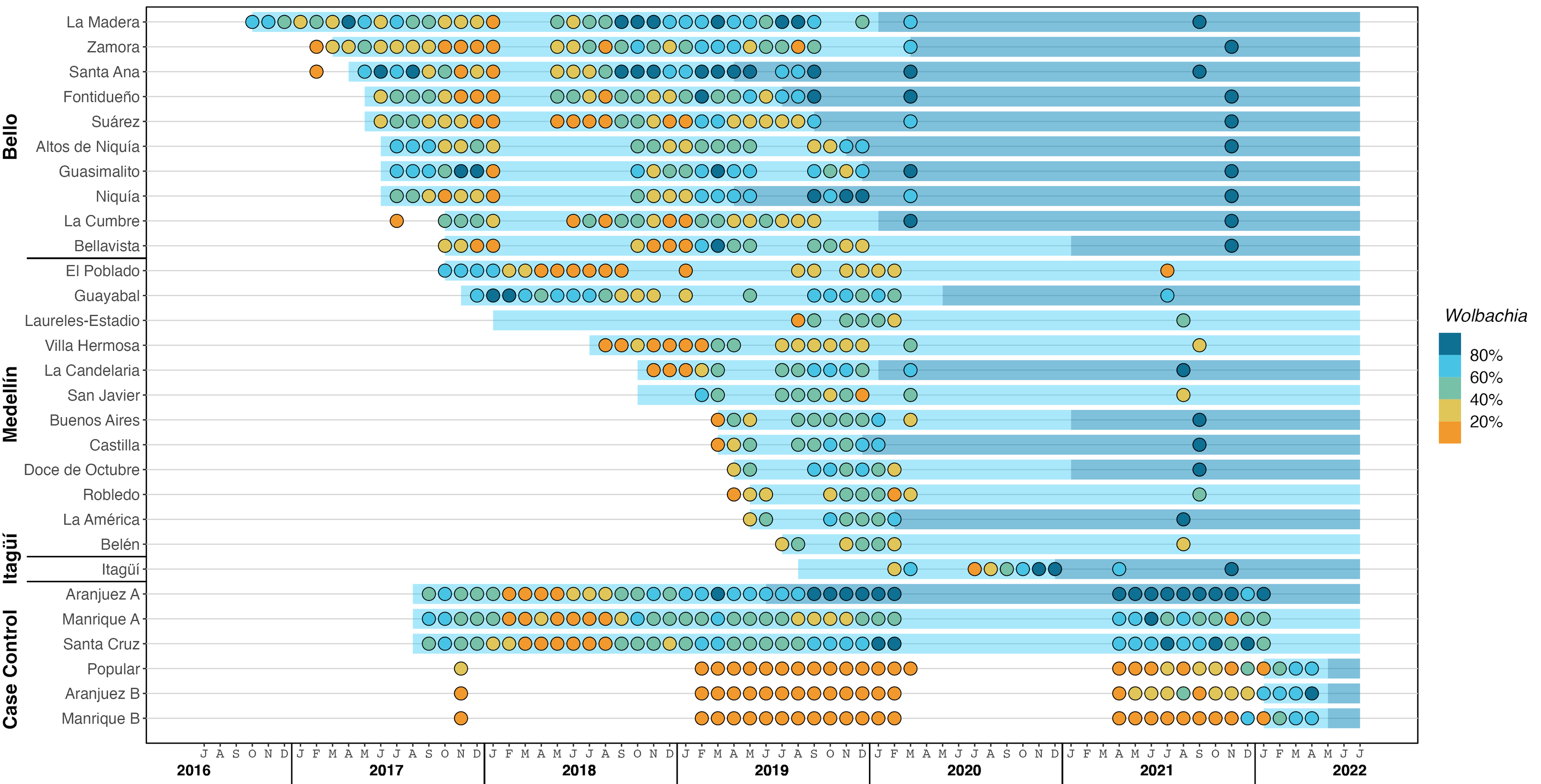

Supplement: S1 Fig — Points indicate the wMel infection prevalence in local Aedes aegypti mosquito populations categorised into levels. Light blue shading indicates the period during which the area is considered ‘partially treated’, commencing from the beginning of wMel releases. Dark blue shading indicates the period during which the area is considered ‘fully treated’, defined as wMel releases completed and wMel prevalence stably at ≥60%. Absence of shading indicates no wMel releases in that area. Itagüí was not disaggregated by commune as wMel was released simultaneously across the whole city. (TIF) [file pntd.0011713.s002.tif]

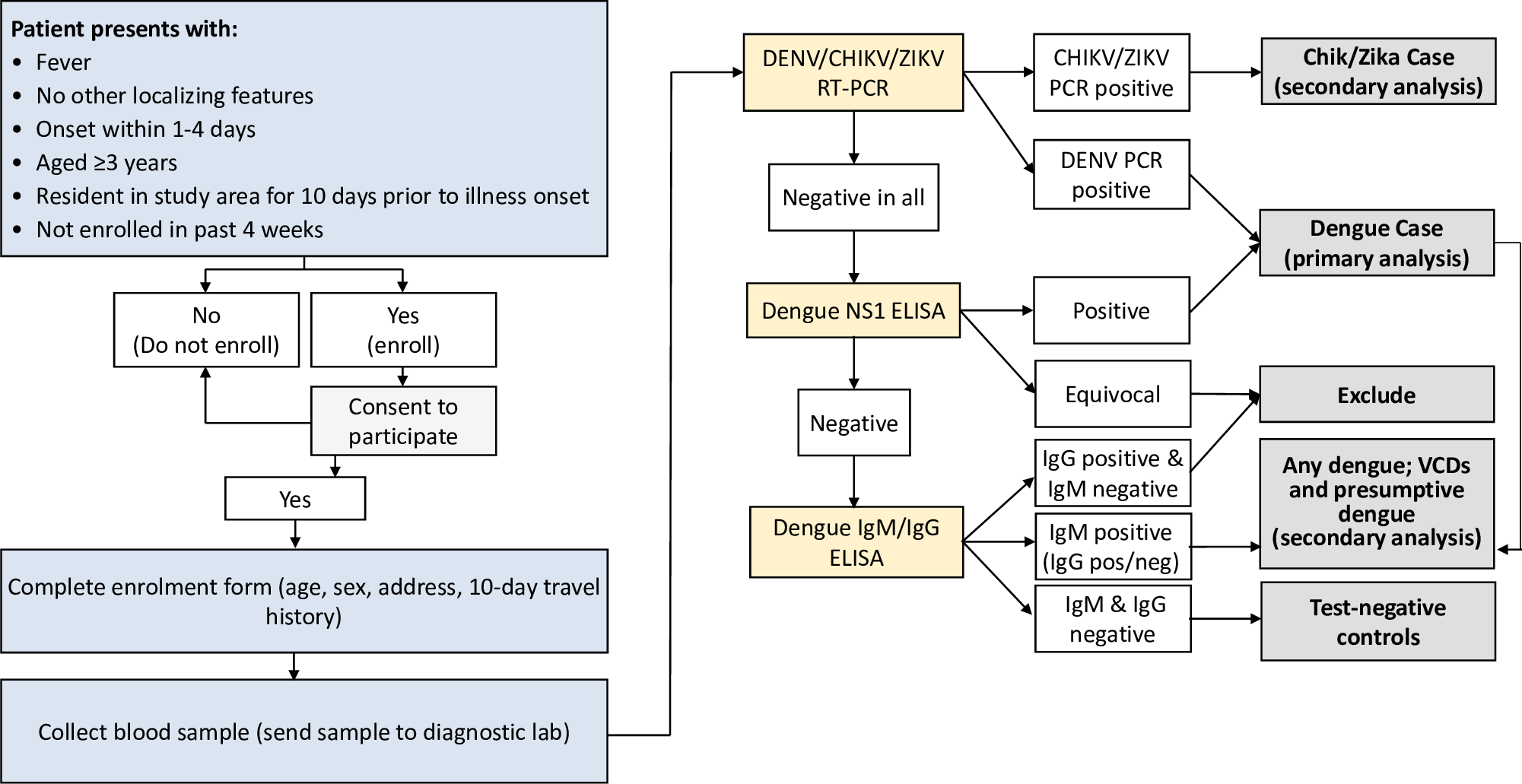

Supplement: S2 Fig — Blue boxes indicate participant recruitment and enrolment activities undertaken at health clinics, including screening against inclusion/exclusion criteria, obtaining written informed consent, and collection of demographic and travel history data and a blood sample. Yellow boxes indicate the laboratory diagnostic testing performed at the project laboratory, the results of which (white boxes) will be used to classify participants as virologically confirmed dengue, presumptive dengue, Zika or chikungunya cases, or arbovirus-negative controls (grey boxes) according to the algorithm shown. (TIF) [file pntd.0011713.s003.tif]

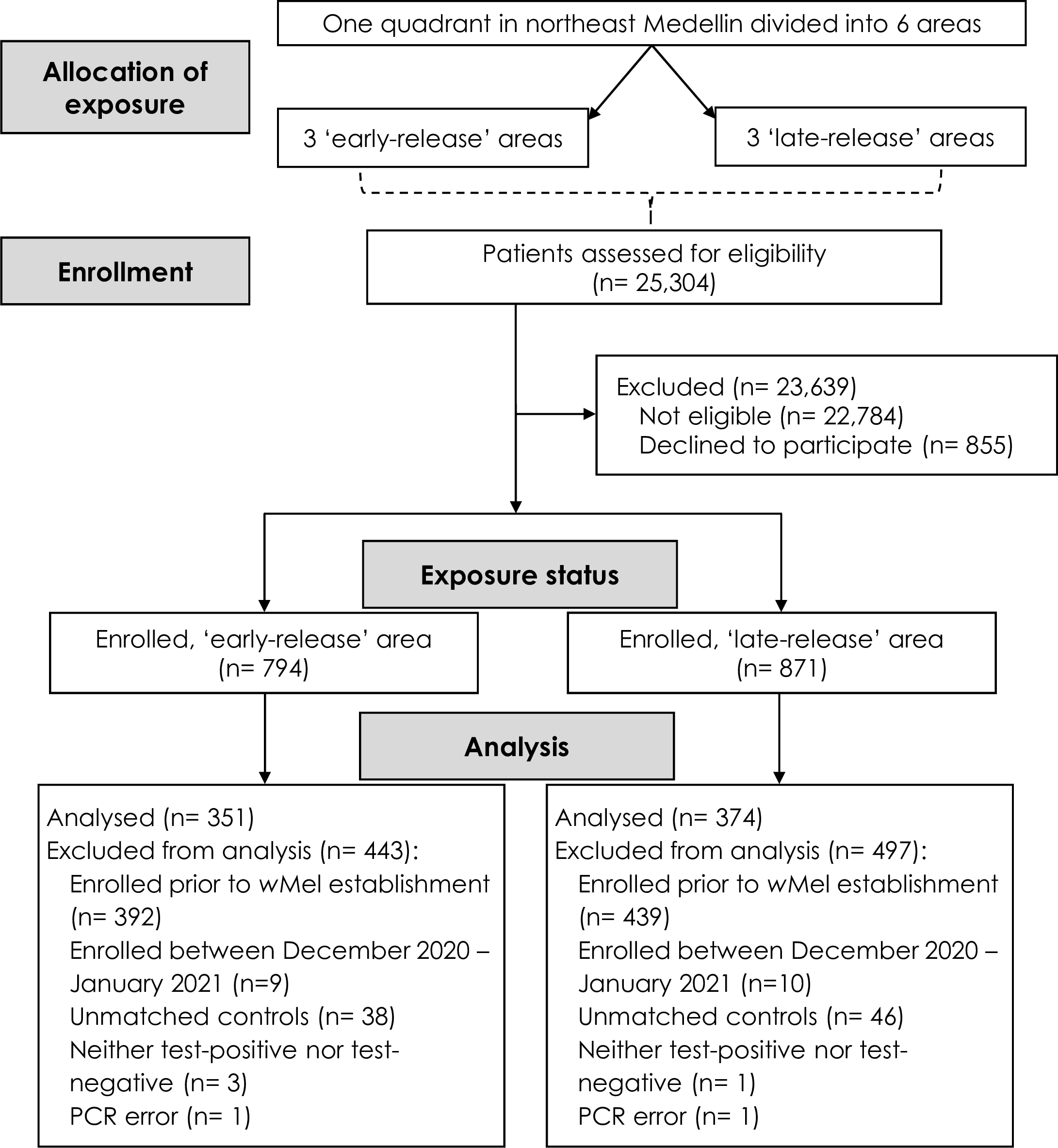

Supplement: S3 Fig — The commonest reasons for exclusion from the analysis dataset were enrolment before the predefined time point of wMel establishment (16 May 2019) and enrolment in a calendar quarter without any VCD and presumptive dengue cases (‘unmatched controls’; July—September 2021). (TIF) [file pntd.0011713.s004.tif]

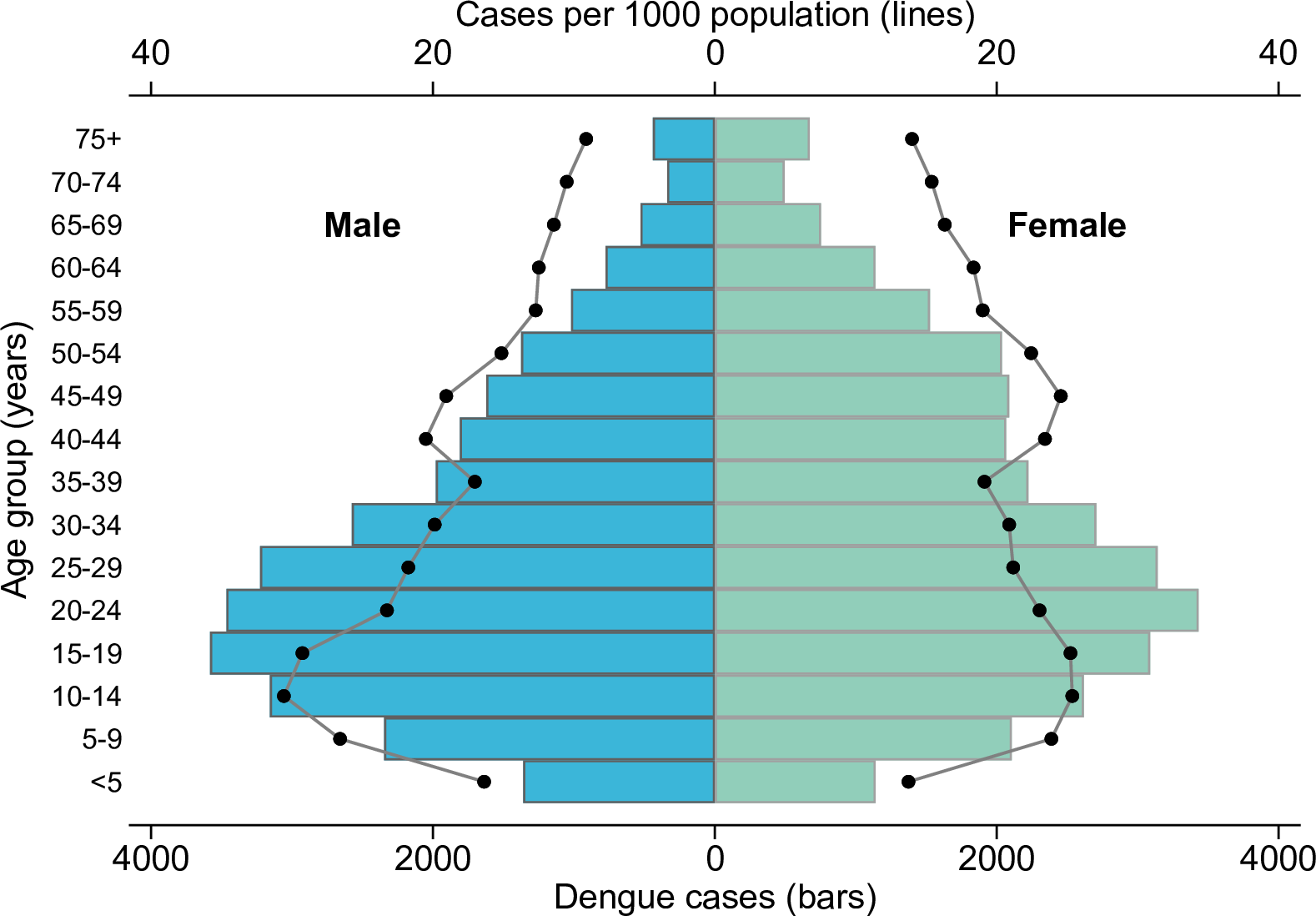

Supplement: S4 Fig — Bars show dengue case numbers and lines show per capita incidence in each five-year age band for males (blue) and females (green), aggregated across the three cities and ten years 2008–2017. Data sources: dengue case data (Instituto Nacional de Salud: http://portalsivigila.ins.gov.co/); age- and sex-specifc population by municipality from 2018 census (DANE: https://sitios.dane.gov.co/cnpv/). (TIF) [file pntd.0011713.s005.tif]
