# Supplementary material for: Reduced dengue incidence following city-wide wMel Wolbachia mosquito releases throughout three Colombian cities: Interrupted time series analysis and a prospective case-control study
Source: PLoS Negl Trop Dis. 2023 Nov 30;17(11):e0011713. doi: 10.1371/journal.pntd.0011713 (PMC10688673; doi:10.1371/journal.pntd.0011713)
Supplement: S1 Appendix — Published 31 March 2022 at https://www.clinicaltrials.gov/study/NCT03631719. (PDF) [file pntd.0011713.s006.pdf]

The impact of deployment of *Wolbachia*-infected mosquitoes on arboviral disease incidence in Medellin, Colombia: a prospective case-control study

Table of Contents

1. Objectives 3
   1.1. Primary Objective 3
   1.2. Secondary Objectives 3
2. Study Design 3
   2.1. Type of Study 3
   2.2. Study area 5
   2.3. Allocation of intervention 5
   2.4. Study Participants 6
   2.5. Duration of Study 6
3. Analysis Endpoints 6
   3.1. Primary Efficacy Endpoint: Virologically-confirmed dengue 6
   3.2. Secondary Efficacy Endpoint: Presumptive dengue, chikungunya and Zika 7
4. Monitoring of *Wolbachia* prevalence in local *Ae. aegypti* populations 7
5. Sample Size Estimation 8
6. Statistical Analysis Method 10
   6.1. General Considerations 10
   6.2. Analysis Sets 10
   6.3. Status of potential participants 11
   6.4. Demographic Characteristics 11

6.5. Analysis Plan for Primary Efficacy Endpoint 11

Intention-to-Treat Analysis 11

Per-protocol analysis 12

6.6. Analysis of Secondary Efficacy Endpoints 13

Impact of Wolbachia deployment on presumptive dengue 13

Impact of Wolbachia deployment on Zika and chikungunya 14

7. Differences between protocol and SAP 14

8. References 15

1. Objectives

1.1. Primary Objective

To assess the efficacy of community-based deployments of *Wolbachia*-infected *Ae. aegypti* mosquitoes in reducing the incidence of symptomatic, virologically-confirmed dengue cases of any severity in Medellin residents aged 3 years and over in *Wolbachia* early-release areas, relative to late-release areas.

1.2. Secondary Objectives

- To measure the efficacy of the *Wolbachia* method in reducing the incidence of symptomatic presumptive dengue (IgM positive) cases
- To measure the efficacy of the *Wolbachia* method in reducing the incidence of symptomatic virologically-confirmed Zika virus and chikungunya virus infection in early-release areas, relative to late-release areas.

2. Study Design

2.1. Type of Study

This is a non-randomised experimental study using a clinic-based prospective case-control design which will be conducted in one quadrant of Medellin, in the northeast of the City (Figure 1). The impact of *Wolbachia* deployments on dengue incidence will be assessed by comparing the exposure distribution (probability of living in a *Wolbachia* early-release vs. late-release area) among virologically-confirmed dengue cases presenting to a network of primary healthcare clinics, against the exposure distribution among patients with febrile illness of non-arboviral aetiology presenting to the same network of clinics in the same temporal windows.

Dengue cases and arbovirus-negative controls are sampled concurrently from within the population of patients presenting with febrile illness to the study clinic network, with case or control status classified retrospectively based on the results of laboratory diagnostic testing. By recruiting participants from within the population of patients presenting to clinics with febrile illness – with dengue test-positive patients classified as cases and test-negative patients classified as controls – the controls are necessarily drawn from the same source

population as the cases, thus avoiding common pitfalls that can introduce selection bias [1]. In this situation, the odds ratio (OR) is an unbiased estimate of the rate ratio in the source population over the period of participant enrolment (the 'risk' period), without the need for any rare disease assumption [2,3].

The null hypothesis is that the relative incidence of virologically-confirmed dengue in *Wolbachia* early-release and late-release areas is one. If *Wolbachia* has a protective effect against DENV transmission, we would expect the relative risk or incidence rate ratio for virologically-confirmed dengue in *Wolbachia* early-release areas compared to late-release areas to be below one.

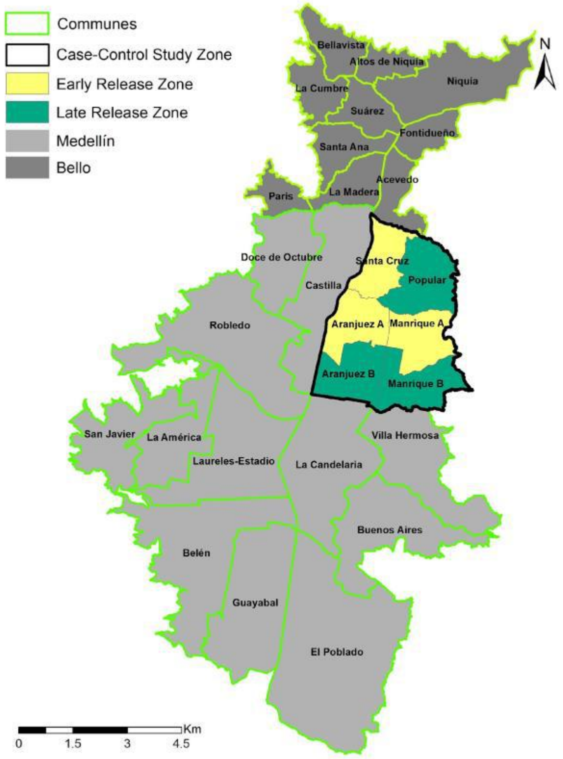

Figure 1. Deployment of Wolbachia across Medellín and Bello with an incident case-control design in a focused study area of 'early' (yellow) and 'late' (green) release zones in the northeast of Medellín. Produced in QGIS version 3.28.3 using administrative boundaries freely available from the three municipal governments: www.medellin.gov.co/geomedellin/datosAbiertos/1043; www.datos.gov.co/Ordenamiento-Territorial/Divisi-n-Pol-tico-Administrativa-Barrios-Bello-Ant/pnhh-ccwd; www.datos.gov.co/Ordenamiento-Territorial/Localizaci-n-Geogr-fica-de-los-Barrios-del-Municip/didi-drqa)

2.2. Study area

The case-control study was conducted only within a focused study area in northeast Medellin, including six contiguous release zones within four comunas (see Figure 1). Among these six release zones, three were allocated as the first zones in Medellin to receive *Wolbachia* deployments, and three as the last. There were no buffer areas between treatment arms, but natural borders (roads, rivers, non-residential areas) were used to define study arm boundaries as much as possible, to limit the spatial spread of *Wolbachia* from treated areas into untreated areas, and of wild-type mosquitoes in *Wolbachia* treated areas. No attempt was made to alter the routine dengue prevention and vector control activities conducted by public and private agencies throughout the study area.

2.3. Allocation of intervention

The allocation of the six zones into two arms was done in a way that maximises balance between the arms with respect to measured factors that may be associated with baseline dengue risk (**Table 1**). Randomised allocation was not feasible, given the small number of zones within the case-control area and the necessarily pragmatic approach to deployments in Medellin in order to achieve large-scale coverage within a short time frame. This time frame also precludes implementing the proposed case-control study across all of Medellin/Bello, given the time required to obtain approvals, establish clinical enrolment processes, and train staff, as well as the requirement to implement a balanced sequence of deployment in the area where the case-control study will be conducted – which would have resource implications for the field entomology and community engagement teams.

Table 1. Allocation of the six release zones into 'early' and 'late' release arms, maximising balance between the two arms in baseline factors that may predict dengue risk

		Ratio of baseline characteristics in Late/Early arms (Ratio of 1 is perfectly balanced)				
'Early' Arm	'Late' Arm	Dengue incidence 2013-2016	Population	Area	%population <15years	Socio-Econ. Status
Aranjuez A<br>Manrique A<br>Santa Cruz	Aranjuez B<br>Manrique B<br>Popular	1.18	0.84	1.09	1.00	1.11

2.4. Study Participants

The study population for measurement of the efficacy endpoint is the population of patients resident in the study area, presenting to the network of participating health clinics with febrile illness, and meeting the eligibility criteria as described in **Table 2**. We enrolled all participants presenting to any of the participating clinics who met the eligibility criteria. Following laboratory testing and classification of participants' diagnostic status, all cases and those controls enrolled within the same calendar quarter as any case will be retained in the dataset for analysis.

Table 2. Participant eligibility criteria

Inclusion criteria	Exclusion criteria
1. Fever (either self-reported or objectively measured, e.g. tympanic membrane temperature ≥38°C) with a date of onset between 1-4 days prior to the day of presentation.	1. Localising features suggestive of a specific diagnosis other than an arboviral infection, e.g. severe diarrhea, otitis, pneumonia.
2. Aged >3 years old.	2. Prior enrollment in the study within the previous 4 weeks.
3. Resided (i.e. slept) in the study area every night for the 10 days preceding illness onset.	

2.5. Duration of Study

Active clinic-based sampling of febrile patients commenced in May 2019 after completion of the 'early' zone *w*Mel releases, with a pause in enrolment between April 2020 – January 2021 due to the COVID-19 pandemic. Enrolment concluded on 31 December 2021, and *w*Mel releases into the 'late' zone are being conducted in January-April 2022.

3. Analysis Endpoints

3.1. Primary Efficacy Endpoint: Virologically-confirmed dengue

The primary outcome measure is virologically-confirmed dengue virus infection in patients reporting febrile illness. Participants are classified as dengue cases for the primary analysis if plasma samples collected 1-4 days after onset of fever test positive for dengue virus nucleic acid by RT-qPCR and/or dengue virus NS1 antigen (BioRad Platelia NS1 ELISA) (see **Figure 2**).

3.2. Secondary Efficacy Endpoint: Presumptive dengue, chikungunya and Zika

A secondary outcome measure of presumptive dengue is used to capture those patients reporting febrile illness who are negative by RT-qPCR and NS1, but who have a positive test for dengue IgM antibody determined using IgM capture ELISA.

Additional secondary outcome measures include chikungunya and Zika virus infection in patients reporting febrile illness. Participants are classified as virologically-confirmed chikungunya cases if chikungunya nucleic acid is detected in plasma samples by RT-qPCR (see **Figure 2**). Participants are classified as virologically-confirmed Zika virus cases if Zika virus nucleic acid is detected in plasma samples by RT-qPCR.

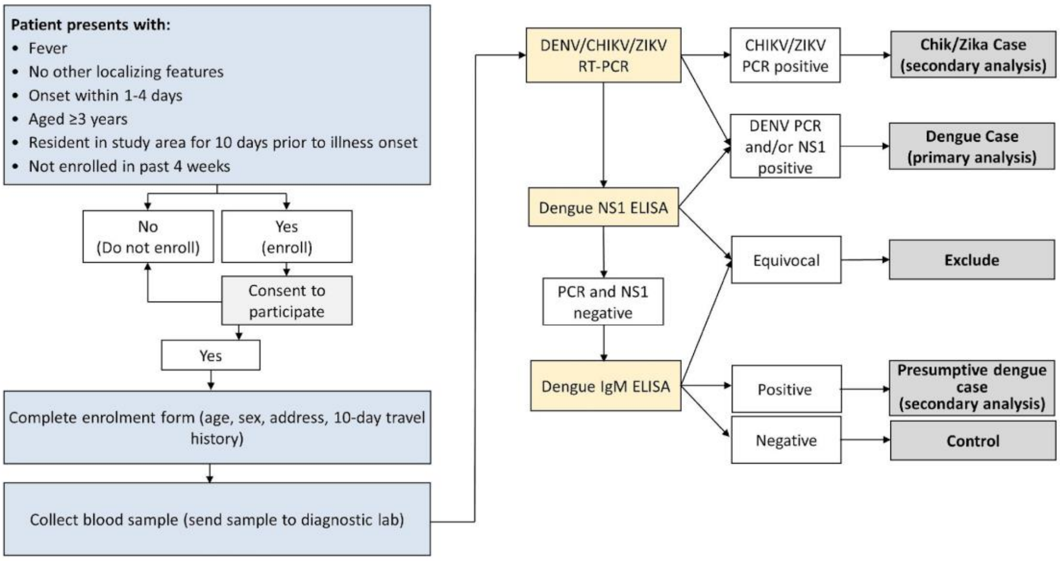

Figure 2. Flowchart of data and sample collection procedures and diagnostic algorithm.

4. Monitoring of *Wolbachia* prevalence in local *Ae. aegypti* populations

Wolbachia prevalence is monitored through a network of BG-Sentinel traps (BioGents) that are evenly spaced throughout the study area at a density of approximately 16 BG traps per km^2. BG traps are serviced weekly, with trapped mosquitoes screened for *Wolbachia* at weekly, fortnightly or monthly intervals throughout the duration of the study, depending on the stage of release and establishment. BG traps that do not catch any mosquitoes in three consecutive weeks are moved to another location. Trapped mosquitoes are identified by

microscopy, and individual *Ae.aegypti* mosquitoes (male and female) are screened using quantitative PCR to detect the presence of *Wolbachia* and to confirm the species as *Ae. aegypti*.

For the purposes of measuring the efficacy endpoint in the primary intention-to-treat analysis, *Wolbachia* will be considered established in each zone after completion of releases in that zone.

5. Sample Size Estimation

It was initially estimated that enrolment of 88 test-positive cases plus four times as many controls would be sufficient to detect a 50% reduction in dengue incidence with 80% power. These sample size estimates were based on standard formulae for calculating sample size/power in a case control study (http://www.openepi.com/SampleSize/SSCC.htm). They aligned with the proposed approach to estimating the intervention effect, which compares the exposure odds among test-positive cases versus test-negative controls, with the null hypothesis that the odds of residence in the *Wolbachia* early-release arm is the same among test-positive cases as test-negative controls, and did not account for clustering of participants in the 3 treated and 3 untreated zones.

A re-evaluation of sample size requirements was conducted in April 2021 following 14 months of participant enrolment (May 2019 – March 2020 and February – April 2021). The re-evaluation found that 27 dengue cases plus four times as many controls would be sufficient to detect an 75% reduction in dengue incidence with 80% power, and 42 cases would be sufficient to detect a 65% reduction in dengue (**Figure 3**, **Table 3**), without accounting for clustering. The AWED trial in Yogyakarta City reported a 77% reduction in virologically-confirmed dengue following *Wolbachia* deployment. High levels of *Wolbachia* were sustained in all intervention clusters throughout the period of active participant enrolment, with a median monthly cluster-level *w*Mel prevalence of 96% (interquartile range [IQR] 91-98%). *Wolbachia* establishment has been more heterogeneous in the early-release areas of this study, with a median monthly area-level *w*Mel of 67% (IQR 43-82%) between May 2019 and March 2020. *Wolbachia* monitoring was paused between April 2020 and April 2021 due to the COVID-19 pandemic. Based on the intervention efficacy reported by the AWED trial, and the *Wolbachia* prevalence observed during the first year of participant enrolment for the

current study, we have chosen to target a 65% reduction in dengue or greater. Clustering was not explicitly accounted for in the sample size calculation, rather an inflation factor of 1.5 is applied to the target sample size and clustering will be controlled for analytically. The revised target sample size is 63 virologically-confirmed dengue cases and at least 252 test-negative controls.

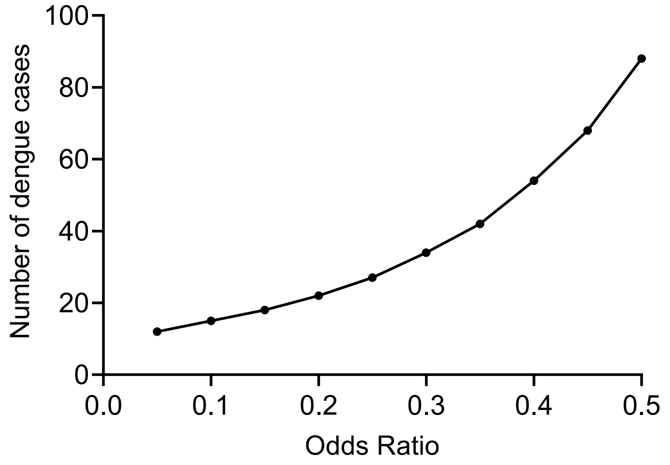

Figure 3. Number of dengue cases required to detect least extreme odds ratio

Table 3. Sample size requirements

Two-sided confidence level (1-alpha)	95	95	95	95	95	95
Power (% chance of detecting true effect)	80	80	80	80	80	80
Ratio of controls to cases	4	4	4	4	4	4
Hypothetical proportion of controls with exposure	50	50	50	50	50	50
Hypothetical proportion of cases with exposure	16.67	20.00	23.08	25.93	28.57	33.33
Least extreme Odds Ratio to be detected (equivalent Risk/Rate Reduction %)	0.20 (80%)	0.25 (75%)	0.30 (70%)	0.35 (65%)	0.40 (60%)	0.50 (50%)
Sample size - cases	22	27	34	42	54	88
Sample size - controls	87	108	134	168	213	352
Total sample size	109	135	168	210	267	440

6. Statistical Analysis Method

6.1. General Considerations

This SAP was developed from the information provided in Protocol version 6.0 dated 02 February 2022.

All statistical analyses will be generated using Stata version 14.0 or higher, or R (R Foundation for Statistical Computing, Austria).

A blinded data review will be conducted to assess the accuracy and completeness of the study database, prior to unblinding of the intervention allocations. The appropriateness of planned statistical analyses will be assessed on a blinded 'dummy' dataset of 50 enrolled participants comprised of exposure and demographic data from 50 randomly selected participants combined with diagnostic results from a separate 50 randomly selected participants. Exposure information and diagnostic results are stored in separate tables within the database. By merging exposure and outcome information from different randomly selected sets of 50 participants we aim to avoid accidental unblinding of the data.

6.2. Analysis Sets

The dataset for analysis will retain all enrolled virologically-confirmed dengue cases, and all test-negative controls that are matched to a case by calendar quarter of enrolment. Controls enrolled outside of the calendar quarters when cases were enrolled will not be used for the primary analysis.

The analysis will be performed on data acquired during the active enrolment period, that is the period commencing when *Wolbachia* is deemed to have been established throughout the early-release area, i.e. following completion of releases in all early-release areas on 16 May 2019. Cases and controls enrolled prior to that date will be excluded from the analysis dataset.

Following a pause in enrolment starting April 2020 due to the COVID-19 pandemic, four out of the 11 clinics were able to resume enrolment in December 2020, with a further five clinics

resuming enrolment in February 2021. As the majority of clinics remained on pause between December 2020 and January 2021, participants enrolled in this period will be excluded from the analysis dataset.

6.3. Status of potential participants

The status of all potential participants that were screened for enrolment will be summarized descriptively, according to the following categories, overall and by treatment arm:

- Number screened
- Number of screened patients that met eligibility criteria
- Number of eligible patients that consented to participate
- Number of consenting participants enrolled in the study
- Number of enrolled participants for whom a blood sample was available for diagnostic testing
- Number of enrolled participants included in datasets for ITT and PP analysis

6.4. Demographic Characteristics

Participants' age and sex will be summarized descriptively overall, and by treatment arm, diagnostic category, and inclusion/exclusion from analysis.

6.5. Analysis Plan for Primary Efficacy Endpoint

Intention-to-Treat Analysis

The intention-to-treat analysis will consider *Wolbachia* exposure as a binary classification based on residence in the early or late-release area. Residence will be defined as the primary place of residence during the 10 days prior to illness onset.

The intervention effect will be estimated from an aggregate OR comparing the exposure odds (residence in the *Wolbachia* early-release area) among test-positive cases versus test-negative controls (for data aggregated across all three release zones within each study arm) with cluster-robust variance estimates. Zone-level averages for age and sex will be included as covariates in the model. The null hypothesis is that the odds of residence in a *Wolbachia* early-release area is the same among test-positive cases as test-negative controls. The resulting OR provides an unbiased estimate of the relative risk providing that the key

assumptions underlying a test negative design are upheld (i.e. that test-negative controls are allowed to include participants who may test positive for dengue at any other time during the study period, and the distribution of non-dengue febrile illness is not associated with the intervention status). Efficacy of the intervention will be calculated as 100*(1-aggregate OR).

Per-protocol analysis

The per-protocol analysis will consider *Wolbachia* exposure as a quantitative index based on measured *Wolbachia* prevalence in local *Ae. aegypti* mosquitoes in the participant's zone of residence, and in locations visited by the participant during the period 3-10 days prior to illness onset. The per-protocol analysis therefore allows for *Wolbachia* exposure to vary in a location over time, and also accounts for human mobility, in terms of the exposure-time that individuals spend outside their area of residence as reported in the travel history interview at enrolment.

Participants are asked about their mobility during the ten days prior to illness onset using a structured interview administered at enrolment. This records the duration of time spent at home, work or school, and other locations visited during daylight hours (5am – 9pm) in the eight-day period. The geographic coordinates of those locations are derived by geo-locating them on a digital map, with the assistance of the respondent. A weighted '*Wolbachia* exposure index' (WEI) will be defined for each participant, as follows. The aggregate *Wolbachia* prevalence for each release area (in both early-release and late-release zones) will be calculated each month from all *Ae. aegypti* trapped in that release area. For the months of February and March 2021, during which participant enrolment, but not *Wolbachia* monitoring, had resumed following a pause due to the COVID-19 pandemic, the area-level *Wolbachia* prevalence for April 2021 will be used. The WEI for each participant will then be calculated by multiplying the area-level Wolbachia prevalence (in the calendar month of participant enrolment) at each of the locations visited, by the proportion of time spent at each location, to give a value on a continuous scale from 0 to 1. For visited locations outside the case-control study area but within Medellin, Bello and Itagui, where city-wide *Wolbachia* releases were undertaken between May 2015 – November 2020, the measured comuna-level *Wolbachia* prevalence will be used. *Wolbachia* monitoring for the city-wide releases was more sporadic with some months without *Wolbachia* screening. For the purpose of the WEI calculation, missing *Wolbachia* frequencies will be interpolated using a regression line

between the last *Wolbachia* monitoring event and the next. Visited locations outside of Medellin, Bello and Itagui will be assumed to have a *Wolbachia* prevalence of zero. The process of calculating WEI will be conducted blinded to participants' case/control status, by partitioning the travel history data from the laboratory diagnostic data, to remove any possibility of observer bias.

An additional per-protocol analysis will be conducted in which the WEI is calculated using only the area-level *Wolbachia* prevalence in the participant's area of residence (in the calendar month of participant enrolment), ignoring the participant's recent travel history. This recognises that dengue exposure risk may be higher at home versus other locations, rather than assuming an even distribution of exposure risk across daytime hours and locations visited.

Cases and controls will be classified by strata of their WEI: 0-0.2; 0.2-0.4; 0.4-0.6; 0.6-0.8; and 0.8-1. This acknowledges that the WEI is not a highly precise measure, and serves to reduce error in exposure classification. The ITT methods described above will be extended to allow for this individual level covariate using a regression approach [4]. A mixed-effects logistic regression will be used, with age and sex as covariates. Zone will be included as a random intercept term and WEI as a random slope term. Efficacy will be calculated as 100*(1-OR). The WEI strata will be included as an unordered covariate to calculate stratum-specific ORs (relative to the baseline 0-0.2 stratum). This approach was selected as model results from the AWED trial [5] suggest a threshold effect of the WEI rather than a 'linear' dose-response relationship. For this reason, the WEI strata was not examined as an ordinal covariate.

6.6. Analysis of Secondary Efficacy Endpoints

Impact of Wolbachia deployment on presumptive dengue

A secondary analysis will estimate the efficacy of *Wolbachia* deployments in reducing symptomatic dengue infection, including virologically-confirmed and presumptive dengue cases. The same intention-to-treat and per-protocol analysis will be used as described for the primary endpoint above, with case populations including participants who are IgM positive as

well as those PCR or NS1 positive for dengue, and with the same control population as for the primary analysis.

Impact of Wolbachia deployment on Zika and chikungunya

There exists no baseline data on the prevalence of Zika or chikungunya infection among febrile patients presenting to primary health care clinics in Medellin, from which to estimate the expected number of cases; therefore, these secondary analyses are exploratory only and not subject to any formal sample size or power calculations. Blood samples from enrolled participants will be tested by Zika and chikungunya PCR for the purpose of defining arbovirus-negative controls for the primary analysis, as described above. These results will permit estimation of the prevalence of virologically confirmed Zika virus and chikungunya virus infection among the study population of ambulatory febrile patients presenting to primary health care.

If ≥20 virologically confirmed Zika or chikungunya cases are detected, a secondary analysis will estimate the efficacy of *Wolbachia* deployments in reducing the incidence of symptomatic virologically confirmed Zika virus and chikungunya virus infection. The same enrolled patient population will be used to analyse all three arbovirus endpoints (dengue, Zika and chikungunya), and the same intention-to-treat and per-protocol analyses will be used as described for the primary (dengue) endpoint above. For Zika and chikungunya, the cases will be defined as enrolled participants who test positive by Zika or chikungunya PCR, respectively, and the controls will be those who test negative to all three arboviruses. If <20 cases of either Zika or chikungunya are detected there will be no formal analysis, only a descriptive analysis of the temporal and spatial distribution of cases.

7. Differences between protocol and SAP

Differences between the approved protocol (version 6.0) and the SAP are listed in the table below.

SAP section, page	Text in SAP	Difference from protocol
5, page 8	The SAP describes a re-evaluation of sample size requirements that was conducted in April 2021, with a revised target sample size.	The protocol only contains the initial sample size calculation.

6.2, page 10	The dataset for analysis will retain all enrolled virologically-confirmed dengue cases, and all test-negative controls that are matched to a case by calendar quarter of enrolment.	The protocol states that "risk sets of cases and controls will be defined by frequency matching enrolled confirmed dengue cases to arbovirus-negative controls with illness onset in the same quarter of the year"
6.5, page 12	For visited locations outside the case-control study area but within Medellin and Bello, where city-wide *Wolbachia* releases were undertaken between May 2015 – October 2019, the measured comuna-level *Wolbachia* prevalence will be used. *Wolbachia* monitoring for the city-wide releases was more sporadic with some months without *Wolbachia* screening. For the purpose of the WEI calculation, missing *Wolbachia* frequencies will be interpolated using a regression line between the last *Wolbachia* monitoring event and the next.	The protocol states that "the measured commune-level *Wolbachia* prevalence from the screening event closest in time to the participant's enrolment will be used"
6.5, page 13	A mixed-effects logistic regression will be used, with age and sex as covariates.	The protocol states that a Cox proportional hazard model will be fitted

8. References

1. De Serres G, Skowronski DM, Wu XW, Ambrose CS. The test-negative design: validity, accuracy and precision of vaccine efficacy estimates compared to the gold standard of randomised placebo-controlled clinical trials. Euro Surveill 2013;18.
2. Greenland S, Thomas DC. On the need for the rare disease assumption in case-control studies. Am J Epidemiol 1982;116:547-53.
3. Vandenbroucke JP, Pearce N. Case-control studies: basic concepts. Int J Epidemiol 2012;41:1480-9.
4. Small DS, Ten Have TR, Rosenbaum PR. Randomization Inference in a Group–Randomized Trial of Treatments for Depression. Journal of the American Statistical Association 2008;103:271-9.
5. Utarini A, Indriani C, Ahmad RA, et al. Efficacy of Wolbachia-Infected Mosquito Deployments for the Control of Dengue. NEJM 2021;384:2177-86.
